# Bufalin Inhibits Tumorigenesis, Stemness, and Epithelial–Mesenchymal Transition in Colorectal Cancer through a C-Kit/Slug Signaling Axis

**DOI:** 10.3390/ijms232113354

**Published:** 2022-11-01

**Authors:** Ling Ding, Yuning Yang, Qin Lu, Dongfeng Qu, Parthasarathy Chandrakesan, Hailan Feng, Hong Chen, Xuzheng Chen, Zhuhui Liao, Jian Du, Zhiyun Cao, Nathaniel Weygant

**Affiliations:** 1 Academy of Integrative Medicine, Fujian University of Traditional Chinese Medicine, Fuzhou 350122, China; 2 Fujian Key Laboratory of Integrative Medicine in Geriatrics, Fujian University of Traditional Chinese Medicine, Fuzhou 350122, China; 3 Key Laboratory of Integrative Medicine, Fujian University of Traditional Chinese Medicine, Fuzhou 350122, China; 4 Department of Medicine, University of Oklahoma Health Sciences Center, Oklahoma City, OK 73104, USA

**Keywords:** patient-derived organoids, personalized therapy, colorectal cancer, cancer stem cells, C-Kit, Slug, bufalin

## Abstract

Colorectal cancer (CRC) is a major source of morbidity and mortality, characterized by intratumoral heterogeneity and the presence of cancer stem cells (CSCs). Bufalin has potent activity against many tumors, but studies of its effect on CRC stemness are limited. We explored bufalin’s function and mechanism using CRC patient-derived organoids (PDOs) and cell lines. In CRC cells, bufalin prevented nuclear translocation of β-catenin and down-regulated CSC markers (CD44, CD133, LGR5), pluripotency factors, and epithelial–mesenchymal transition (EMT) markers (N-Cadherin, Slug, ZEB1). Functionally, bufalin inhibited CRC spheroid formation, aldehyde dehydrogenase activity, migration, and invasion. Network analysis identified a C-Kit/Slug signaling axis accounting for bufalin’s anti-stemness activity. Bufalin treatment significantly downregulated C-Kit, as predicted. Furthermore, overexpression of C-Kit induced Slug expression, spheroid formation, and bufalin resistance. Similarly, overexpression of Slug resulted in increased expression of C-Kit and identical functional effects, demonstrating a pro-stemness feedback loop. For further study, we established PDOs from diagnostic colonoscopy. Bufalin differentially inhibited PDO growth and proliferation, induced apoptosis, restored E-cadherin, and downregulated CSC markers CD133 and C-Myc, dependent on C-Kit/Slug. These findings suggest that the C-Kit/Slug axis plays a pivotal role in regulating CRC stemness, and reveal that targeting this axis can inhibit CRC growth and progression.

## 1. Introduction

Colorectal cancer (CRC) is the second leading cause of cancer-associated mortality globally. Although advances in screening allow for early diagnosis of CRC and potentially curative therapy, metastatic CRC is often accompanied by drug resistance and a poor 5-year survival rate of 12% [1]. Heterogeneity is considered to be a primary reason for poor prognosis in CRC [2], and mounting evidence links this aspect of the tumor epithelia to cancer stem cells (CSCs) [3,4]. CSCs are a subpopulation of epithelial cells with stem cell characteristics enabling them to produce progeny that can retain self-renewal ability as well as differentiate into cancer cells with various phenotypes, which is a direct cause of tumorigenesis [5]. Dynamic tumor invasion and metastasis processes require the cooperation of multiple cell types in the tumor microenvironment [6,7]. Within the tumor microenvironment, the niche in which CSCs reside enables cell plasticity and enhances metastatic potential, which is closely related to the process of epithelial–mesenchymal transition (EMT)—a continuous, dynamic process under the control of a set of specialized transcription factors, which activates the transformation of tumor epithelial cells into mesenchymal-like cells, thereby promoting the invasive characteristics of tumor cells [8].

Previous studies have shown that CSCs and intestinal stem cells (ISCs) have similar regulatory mechanisms, including Wnt/β-catenin, NOTCH and other signaling pathways and markers such as CD44, CD133, ALDH1 and LGR5 [9,10]. A bottom–up model describes transit-amplifying cells generated from LGR5+ ISCs in the epithelial crypt differentiating into other epithelial cell types and then gradually migrating to the tip of the villus [11]. Since the successful construction of murine CRC organoids from a single LGR5+ crypt stem cell by the Hans Clevers team, organoid culture techniques have become increasingly mature [12], and specialized organoid culture conditions for adenoma, adenocarcinoma, Barrett’s esophagus, and other cancerous and precancerous epithelia have been explored [13,14,15]. In CRC, patient-derived organoids (PDOs) can completely reproduce epithelial structure and molecular characteristics, including the heterogeneity of tumors. This individualized model more accurately reflects the true response of patients to drug treatment and is starting to be applied in drug screening [16,17,18].

C-Kit (CD117) is a transmembrane receptor tyrosine kinase encoded from the KIT gene. C-Kit is associated with drug resistance and metastasis in ovarian cancer, salivary adenoid cystic tumor, prostate cancer, and other tumors [19]. Overexpression or mutation of C-Kit promotes aggressive properties in tumor cells, including growth, pro-survival/anti-apoptotic signaling, and migration. C-Kit has also been shown to regulate the differentiation and survival of CSCs. In non-small cell lung cancer (NSCLC), tumor cells expressing C-Kit demonstrate increased self-renewal and chemoresistance [20], and there have been similar findings in ovarian and prostate cancer models [21,22]. Although the role of C-Kit in CRC CSCs has not been explored extensively, there are interesting clues as to its role in the colonic ISC/CSC niche. Rothenberg et al. found that C-Kit+ cells are interspersed with LGR5+ stem cells at the base of the colonic crypt, and that most C-Kit+ colonic epithelial cells are CD44-positive. Furthermore, C-Kit/CD44 double-positive colonic epithelial cells are capable of promoting the formation of Lgr5+ organoids, suggesting an important supportive role in the ISC/CSC niche [23]. Slug, a key EMT transcription factor encoded by the SNAI2 gene, is closely associated with tumor progression and can be used as a potential marker of lymph node metastasis and poor prognosis in CRC [24]. In basal breast cancer, expression of Slug enriches CD44+/CD24- stem-like populations [25]. Furthermore, Slug may act in a positive feedback loop mechanism with C-Kit in mesothelioma and NSCLC [26,27]. Combined these findings suggest the potential value of further exploring the relationship between C-Kit and Slug and their role in the context of CRC and its epithelial niche. 

Bufalin is a cardiotonic steroid found in toad venom, along with the structurally related component cinobufagin, which is used to treat various cancers in Mainland China [28]. Bufalin has antiproliferative and pro-apoptotic effects on various tumor cells including liver, colorectal, osteosarcoma, lung, and others [29,30,31,32]. Furthermore, bufalin can inhibit colitis and APC mutation-associated CRC tumorigenesis in murine models [33]. In addition to inhibiting proliferation and promoting apoptosis through apoptosis-related genes [28,34], bufalin’s therapeutic effect on CRC may also be related to regulating stemness-associated factors and multidrug resistance. However, current research is limited, and further exploration of the mechanism of bufalin against CRC stemness is needed. Herein, we utilized in vitro CRC cell lines and ex vivo patient-derived organoid models to clarify the function and mechanism of bufalin in CRC. 

## 2. Results

### 2.1. Bufalin Inhibits the Proliferation and Viability of CRC Cells

In order to investigate the effect of bufalin on the proliferation of CRC cells, MTT assay was performed following 72 h of treatment. IC_50_ values were calculated for each cell line and revealed that low nanomolar concentrations of bufalin can significantly inhibit the proliferation of CRC cells at this timepoint, with HCT116 showing the most sensitivity (IC_50_ = 1.661 nM), followed by DLD1 (IC_50_ = 4.279 nM) and SW480 (IC_50_ = 30.03 nM) (Figure S1A). To assess the impact on cell viability, we performed LIVE/DEAD (Calcein-AM/propidium iodide) staining after 48 h of treatment, which showed that bufalin inhibits growth and survival in all three CRC cell lines, with more pronounced effects apparent at 25 nM concentrations and higher. The increase in propidium iodide-stained HCT116 cells was also notable, confirming the high sensitivity of this cell line to bufalin (Figure S1B).

### 2.2. Bufalin Inhibits Epithelial–Mesenchymal Transition and Invasiveness of CRC Cells

**Figure 1:**
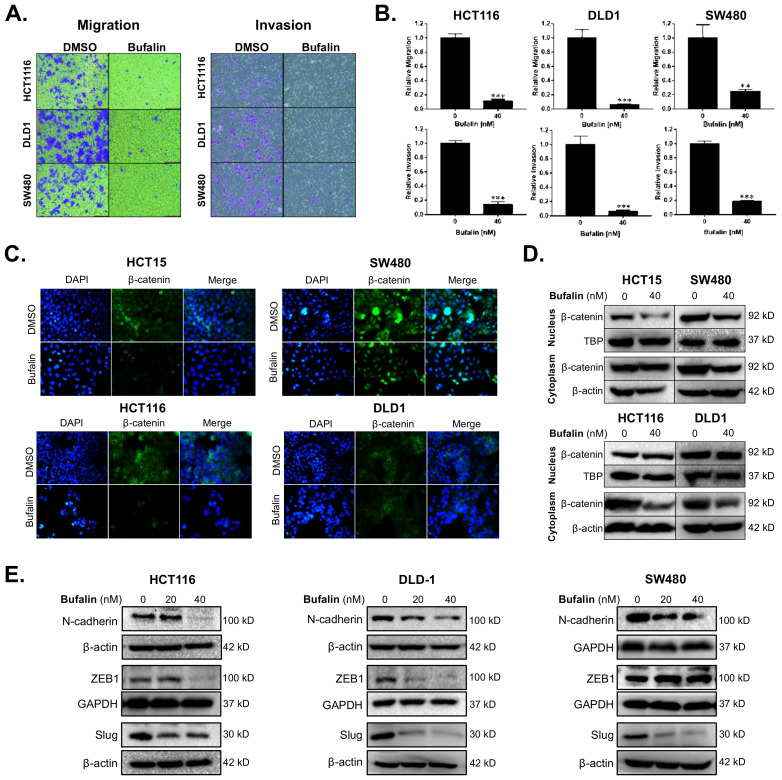
Bufalin inhibits epithelial–mesenchymal transition, β-catenin nuclear translocation, and metastatic properties of colorectal cancer cells. (**A**) Representative images of transwell migration and invasion assays for HCT116, DLD1 and SW480 cells after 48 h of treatment with 40 nM bufalin. (**B**) Quantitation of transwell migration and invasion assays revealing that 40 nM bufalin has a significant inhibitory effect on pro-metastatic migratory and invasive properties in conditions of chemotaxis for HCT116, DLD1, and SW480 CRC cells. (**C**) Immunofluorescence staining showing reduced translocation of nuclear β-catenin in HCT15 and SW480 cell lines after 48 h of 40 nM bufalin treatment and reduced cytoplasmic expression of β-catenin in HCT116 and DLD1 cell lines, which did not show visual evidence of nuclear translocation at baseline, after 24 h of 40 nM bufalin treatment. (**D**) Western blot results demonstrating reduced nuclear β-catenin expression in HCT15 and SW480 CRC cells, and reduced cytoplasmic β-catenin expression in SW480, HCT116, and DLD1 CRC cells after 40 nM bufalin treatment. (**E**) Western blot results showing that bufalin potently inhibits the expression of EMT marker N-cadherin and EMT transcription factors ZEB1 and Slug (** *p* < 0.01, *** *p* < 0.001, images at 10× magnification).

Migration and invasion are important features of malignant tumors and also the premise for tumor metastasis. The anti-metastatic activity of bufalin was examined by transwell migration and invasion assays under conditions of chemotaxis. Compared with the vehicle control group, migratory cells of the bufalin treated group were reduced by approximately 88–93% in HCT116, 90–98% in DLD1, and 65–80% in SW480 (HCT116: *p* < 0.0001; DLD1: *p* = 0.0002; SW480: *p* = 0.0072) (Figure 1A,B). The ability for tumor cells to break through basement membrane is a hallmark of invasion and important basis for malignant tumor diffusion [35]. Transwell invasion assay was conducted to further study the ability of CRC cells to break through the basement membrane after bufalin treatment. At 48 h, compared with the control group, the invading cells in the bufalin treated group were reduced by 80–98% in HCT116, 90–97% in DLD1, and 78–85% in SW480 (HCT116: *p* < 0.0001; DLD1: *p* = 0.0002; SW480: *p* < 0.0001) (Figure 1A,B). These findings demonstrate that bufalin functionally inhibits CRC cell migration and invasion. 

Aberrant expression of β-catenin leads to instability in the complex formed with E-cadherin, resulting in a loss of epithelial characteristics and an increasingly invasive phenotype. This is further supported by nuclear translocation of β-catenin leading to EMT gene expression [36]. To determine the expression levels and location of β-catenin in CRC cells after bufalin treatment, immunofluorescence and Western blot assays were performed. Under normal conditions, the HCT15 and SW480 cell lines showed frequent nuclear β-catenin, while HCT116 and DLD1 demonstrated mainly cytoplasmic expression of β-catenin. Treatment with 40 nM bufalin decreased the nuclear localization of β-catenin in HCT15 and SW480 cells (Figure 1C). The intensity of β-catenin staining also decreased in the cytoplasm of SW480, HCT116, and DLD1 cells after treatment (Figure 1C). Furthermore, the expression levels of β-catenin in isolated cytoplasmic or nuclear components were analyzed by Western blot and decreased after bufalin treatment, consistent with the results of the immunofluorescence experiment (Figure 1D). To determine the effect of bufalin on EMT factors that may be downstream of β-catenin, we performed additional immunoblot assays. Treatment with 20 or 40 nM of bufalin was able to significantly down-regulate the expression of mesenchymal marker N-cadherin and key EMT transcription factor Slug in HCT116, DLD1, and SW480 cell lines. Bufalin treatment also led to downregulation of the ZEB1 EMT transcription factor in HCT116 and DLD1, but not SW480 cells (Figure 1E). These findings demonstrate that bufalin has a potent inhibitory effect on EMT and the metastatic characteristics of CRC.

### 2.3. Bufalin Inhibits Stemness in CRC Cell Lines

**Figure 2:**
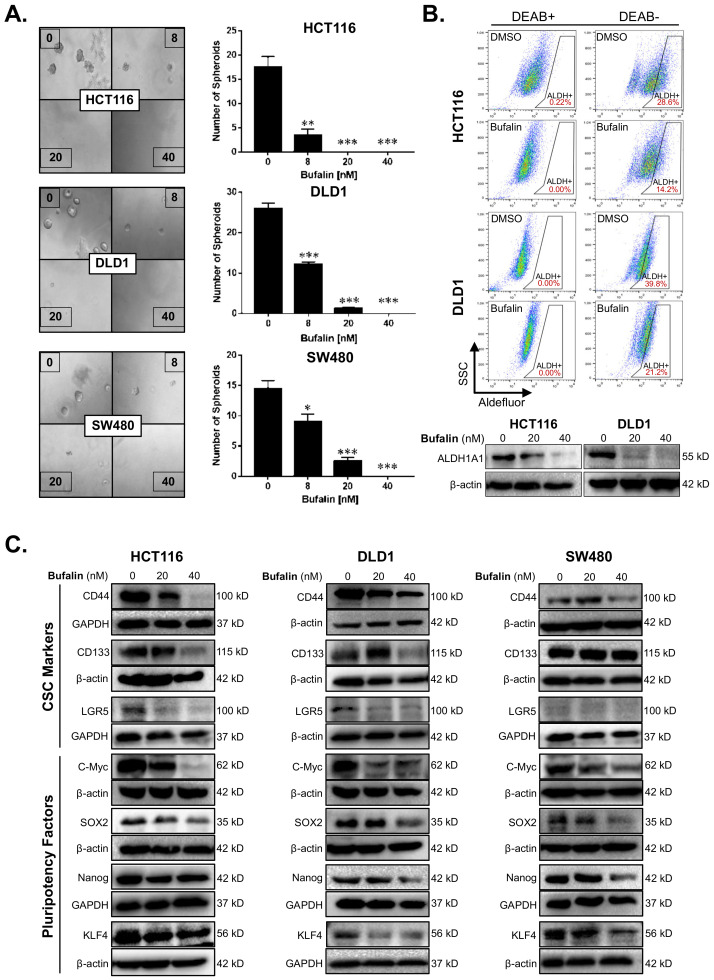
Bufalin inhibits functional stemness, aldehyde dehydrogenase activity, and the expression of CSC/pluripotency markers in colorectal cancer cells. (**A**) Matrigel spheroid formation assay results showing that treatment with 8–40 nM bufalin can significantly reduce the number of spheroids formed in HCT116, DLD1, and SW480 cell lines. (**B**) The enzyme activity of ALDH was detected by flow cytometry using DEAB as a gating control, revealing that treatment with 40 nM bufalin significantly reduces ALDH+ HCT116 and DLD1 cells, which was confirmed by Western blot. (**C**) Bufalin inhibits the expression of CSC markers CD44, CD133, and LGR5, as well as the pluripotency factors C-Myc, SOX2, Nanog, and KLF4 in a cell line-dependent manner (* *p* < 0.05, ** *p* < 0.01, *** *p* < 0.001, images at 10× magnification).

The sphere-forming ability of individual tumor cells in a low growth factor, 3D culture environment reflects the stemness of the tumor [37]. We performed a matrigel spheroid formation assay to detect CRC stemness after bufalin treatment. Bufalin significantly inhibited the ability of CRC cells to form spheroids compared with DMSO vehicle controls (*p* < 0.01) (Figure 2A). Acetaldehyde dehydrogenase (ALDH) converts acetaldehyde to acetic acid in the human body, thus maintaining cell homeostasis, which is particularly important for the self-renewal ability of stem cells and CSCs [38]. ALDEFLUOR assay, in which the fluorescence intensity is proportional to the activity of ALDH, was performed by flow cytometry using HCT116 and DLD1 cells. Diethylaminobenzaldehyde (DEAB), which can inhibit the activity of ALDH, was used as a negative control to obtain background readings. The activity of ALDH enzymes decreased from 28.6% to 14.2% and 39.8% to 21.2% for the HCT116 and DLD1 cell lines, respectively, after 24 h of bufalin treatment. Consistent with these findings, key CSC-related ALDH subunit ALDH1A1 expression levels were decreased in both lines after 20 or 40 nM bufalin treatment (Figure 2B). Furthermore, treatment of SW480 cells with bufalin also resulted in a similar decrease in ALDH1A1 expression (Figure S2A,B).

To further investigate the molecular drivers of bufalin’s anti-stemness activity, we performed additional immunoblotting analyses for key CSC targets. Previous studies suggest that bufalin can target CD44 and CD133+ CSCs, as well as affect the expression of these and other functional CSC markers [39,40,41]. Treatment with 40 nM bufalin resulted in downregulation of CD44 in HCT116, DLD1, and SW480 cell lines. However, CD133 and LGR5 levels were only significantly downregulated in the HCT116 and DLD1 cell lines (Figure 2C). Pluripotency is a key trait of stem cells and is controlled on the molecular level by the expression of a cassette of transcription factors: C-Myc, SOX2, KLF4, and Nanog [42]. Bufalin treatment led to downregulation of all four pluripotency factors in all cell lines tested after 40 nM of bufalin treatment (Figure 2C). Together, these findings demonstrate bufalin’s anti-stemness activity in CRC cells.

### 2.4. Bufalin’s Anti-CSC Activity Is Dependent on a C-Kit/Slug Signaling Axis

**Figure 3:**
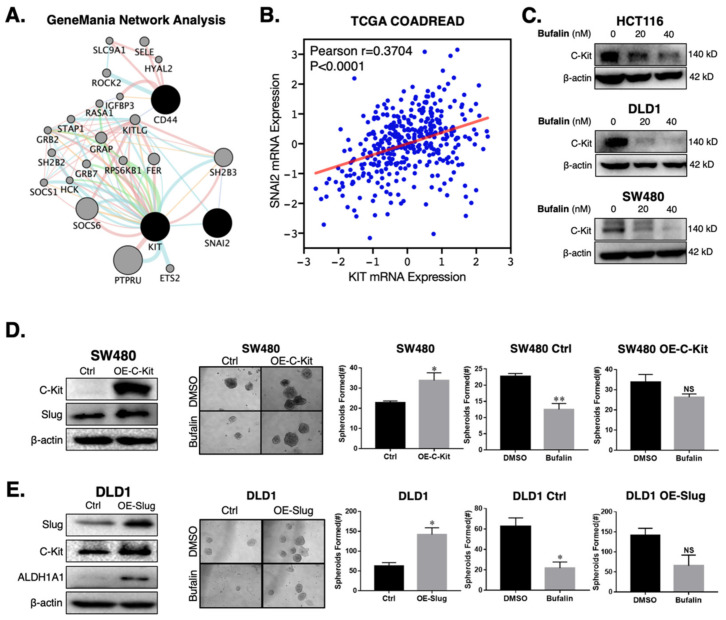
C-Kit and Slug form a positive-feedback signaling axis in CRC, which is a target of bufalin’s anti-stemness activity. (**A**) GeneMANIA network analysis showing that C-Kit (KIT) and Slug (SNAI2) are pathway interactors. (**B**) Correlation analysis from TCGA’s COADREAD RNA-Seq dataset demonstrating that the mRNA expression of C-Kit and Slug are positively correlated (Pearson r = 0.3704, *p* < 0.0001). (**C**) Immunoblotting results confirming that C-Kit is strongly downregulated following 20 or 40 nM bufalin treatment in HCT116, DLD1 and SW480 cell lines. (**D**) Overexpression of C-Kit (OE-C-Kit) in the SW480 cell line results in up-regulation of Slug, increased matrigel spheroid formation capacity at baseline (~50% increase; *p* < 0.05), and resistance to bufalin’s anti-stemness activity as evidenced by an approximately 50% reduction in vector control spheroids (SW480 Ctrl; *p* < 0.01) compared to a <20% reduction in OE-C-Kit spheroids (SW480 OE-C-Kit; NS). (**E**) Slug over-expression (OE-Slug) in the DLD1 cell line results in upregulation of C-Kit and ALDH1A1, increased matrigel spheroid formation capacity at baseline (>100% increase; *p* < 0.05), and resistance to bufalin’s anti-stemness activity as evidenced by an approximately 66% reduction in vector control spheroids (DLD1 Ctrl; *p* < 0.05) compared with a statistically insignificant reduction in OE-C-Kit spheroids (DLD1 OE-C-Kit; NS). (*p < 0.05, ** *p* < 0.01, NS = Not Significant).

Given the consistent down-regulation of the CD44 CSC marker and Slug EMT transcription factor in the three CRC cell lines studied, we performed network analysis to identify potential interactors that could connect these two highly interrelated processes [43,44,45]. The network analysis revealed the connectivity between CD44, the receptor tyrosine kinase receptor C-Kit (KIT), and Slug (SNAI2) (Figure 3A). Analysis of the Cancer Genome Atlas Colorectal Cancer (COADREAD) RNA-Seq data set showed a significant positive relationship between KIT and SNAI2 mRNA expression (*n* = 380, Pearson r = 0.3704, *p* < 0.0001) (Figure 3B). To determine whether bufalin has activity against C-Kit, we performed immunoblotting for the three CRC cell lines after treatment with 20 or 40 nM bufalin. Similarly to Slug (Figure 1E), C-Kit expression was consistently down-regulated in all lines at 20 and 40 nM concentrations (Figure 3C and Figure S2C). These findings demonstrate the relationship between C-Kit and Slug in human CRC, which are both sensitively affected by bufalin treatment.

In order to determine if there is a direct mechanistic relationship between C-Kit and Slug which affects bufalin’s therapeutic efficacy, we overexpressed C-Kit in the SW480 cell line which has low endogenous C-Kit expression. C-Kit overexpression was confirmed by Western blotting (Figure 3D) and qPCR (Figure S2D) and led to an approximately 400% increase in Slug expression at the mRNA (Figure S2E) and protein levels (Figure 3D). Functionally, C-Kit overexpression promoted stemness in CRC and significant resistance to bufalin’s anti-stemness effect in matrigel spheroid assay (*p* = 0.0306; Figure 3D) To ascertain the role of Slug in this process, we overexpressed Slug in the DLD1 cell line, which has low endogenous expression of this protein and a more epithelial appearance compared to HCT116 and SW480 cell lines, which have previously been classified in the high EMT CMS4 CRC subtype [46]. Overexpression of Slug in DLD1 cells resulted in >2 fold upregulation of C-Kit expression, a significant increase in the number of matrigel spheroids formed (*p* = 0.006) and significant resistance to bufalin (Figure 3E). Furthermore, Western blot results showed that CD44 and ALDH1A1 levels increased significantly in cells overexpressing Slug (Figure 3E and Figure S2F). Taken together, these findings provide evidence that C-Kit and Slug signaling act in the manner of a positive feedback loop promoting stemness in CRC, which can be targeted using bufalin.

### 2.5. Bufalin Inhibits C-Kit/Slug Signaling and Tumorigenesis in CRC Patient-Derived Organoids

**Figure 4:**
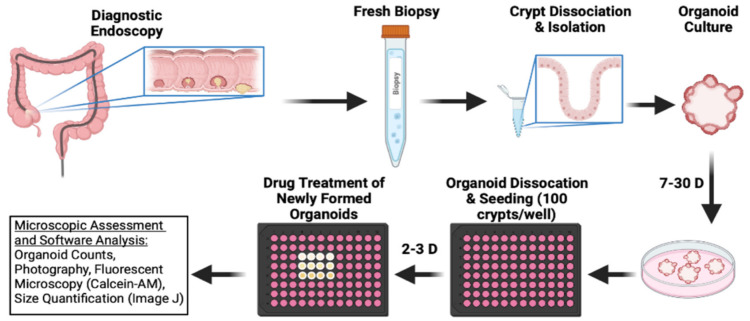
Schematic outline of CRC patient-derived organoid establishment and treatment procedure used to further ascertain bufalin’s activity against C-Kit/Slug signaling.

To test the efficacy of bufalin in clinically relevant models of CRC, we obtained endoscopy samples from patients undergoing diagnostic procedures, isolated epithelial crypts, and cultured patient-derived organoids to maturity (Figure 4). In total, five PDO models were established for this study (T0519, T0528, T0714, T1208, and T1101). Details and clinical marker expression for PDOs are provided in Table 1. After PDO establishment, mature PDOs were digested, seeded into 96-well plates at a density of 100 crypts per well, and cultured until mature organoids formed. After reaching maturity, the PDOs were treated with 0 (DMSO), 20, or 40 nM bufalin and the response was monitored. 

**Figure 5:**
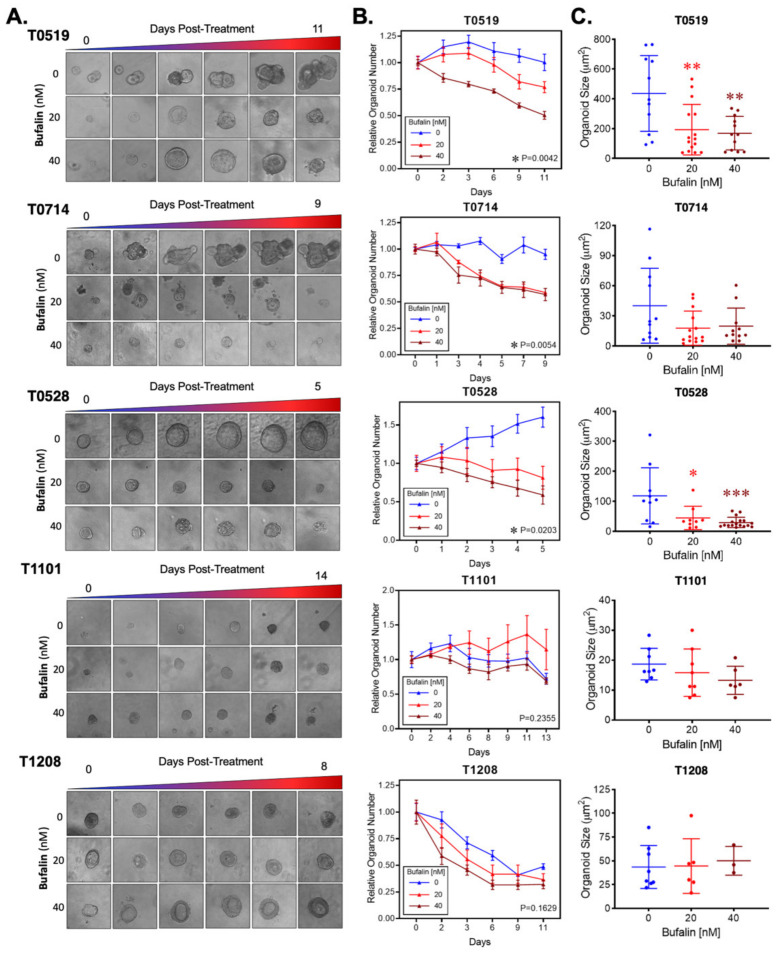
Bufalin inhibits tumorigenesis in organoids derived from patients with colorectal adenoma and adenocarcinoma. (**A**). Representative microscopy images showing the effect of 20 or 40 nM bufalin treatment on patient-derived organoids of colorectal adenoma (T0519), colon adenocarcinoma (T0528), and rectal adenocarcinoma (T0714, T1208, and T1101) patient-derived organoids. (**B**) Quantification of the time-dependent change in organoid number over the course of bufalin treatment in five PDOs demonstrating significant decreases in the number of organoids for T0519, T0528, and T0714. (**C**) Quantification of the final organoid sizes determined by microscopy measurements in the five PDOs, demonstrating a significant reduction in the size of T0519 and T0528, but no statistically significant reduction in T0714, T1208, and T1101. (* *p* < 0.05, ** *p* < 0.01, *** *p* < 0.001). (Images at 20× magnification).

**Figure 6:**
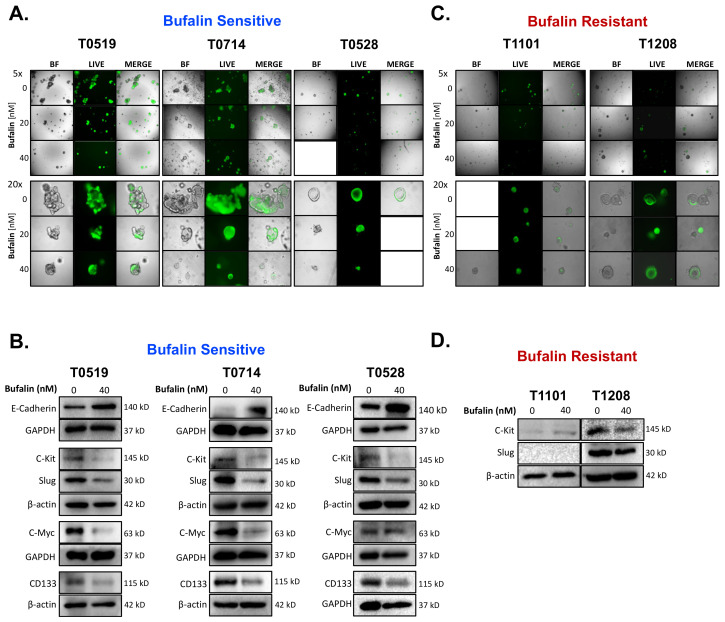
Bufalin-sensitive CRC PDOs are characterized by strong inhibition of C-Kit/Slug, restoration of E-cadherin, and decreases in CD133 and C-Myc expression. (**A**) Representative images of Calcein-AM staining of T0519, T0528, and T0714 at the termination of bufalin treatment, confirming the decrease in live PDOs at 20 and 40 nM concentrations. (**B**) Immunoblotting of PDO lysates demonstrating a strong decrease in the expression of both mechanistic targets C-Kit and Slug, decreased expression of CSC markers CD133 and C-Myc, and upregulation of epithelial marker E-Cadherin following 48 h of treatment with 40 nM of bufalin compared to vehicle control in PDOs sensitive to bufalin T0519, T0528 and T0714. (**C**) Representative images of Calcein-AM staining of T1208 and T1101 that confirm that there are no significant changes in the number or size of live PDOs at the end point of bufalin treatment. (**D**) Immunoblotting of PDO lysates demonstrating little or no effect on C-Kit and/or Slug protein expression after 48 h of treatment with 40 nM bufalin compared to vehicle control in bufalin-resistant PDOs T1208 and 1101. (A-C Images: Top panels: 5× magnification; Bottom panels: 20× magnification).

Bufalin significantly inhibited the growth and survival of three PDOs (T0519, T0528, and T0714), as determined by reductions in the numbers (T0519: *p* = 0.0042, T0528: *p* = 0.0203, T0714: *p* = 0.0054) and sizes of PDOs (T0519 and 0528; *p* < 0.05) (Figure 5A–C). However, two PDOs, T1208 and T1101, were not sensitive to bufalin treatment, revealing a limited inhibitory effect even with a relatively longer duration of treatment (up to 13 days) (Figure 5A–C). In PDOs that demonstrate bufalin sensitivity (T0519, T0528, and T0714), morphological changes also suggested antitumor activity, including structural fracture of PDOs and the formation of characteristic apoptotic membrane blebbing (Figure 5A and Figure 6A). Comparatively, bufalin-resistant organoids demonstrated less of these features (Figure 5A and Figure 6C). In confirmation of the reduction in tumorigenesis, Calcein-AM live cell staining at the end of the organoid growth assay also confirmed the reduction of the number of living PDOs in bufalin sensitive, but not bufalin-resistant PDOs (Figure 6A,C). In support of the proposed mechanism of bufalin activity, treatment of PDOs led to downregulation of C-Kit and Slug proteins, which was accompanied by a decrease in the expression of CD133 and C-Myc in PDOs sensitive to bufalin. Furthermore, up-regulation of E-cadherin was observed, suggesting the potential for anti-EMT effects, in these sensitive organoids (Figure 6B). In contrast, C-Kit and Slug were not significantly affected in bufalin-resistant PDOs T1208 and T1101, with T1101 showing a slight increase in C-Kit and no expression of Slug, and T1208 showing only slight decreases in both markers (Figure 6D). Finally, we analyzed available serum marker levels (CEA, CA19-9, CA-125, and AFP) from the five patient donors and found that serum levels of CA19-9 and AFP were relatively elevated in resistant compared to sensitive PDOs, although none of the analyte levels reached the clinical significance level (Table 1). In general, these findings demonstrate variable responses to bufalin in organoids derived from patients with CRC, which are likely to depend on the activity of the C-Kit/Slug signaling axis.

## 3. Discussion

Our findings demonstrate that bufalin can significantly inhibit colorectal cancer stemness and tumorigenesis, which is accompanied by its activity against EMT markers (Slug, ZEB1, N-Cadherin) and functional stem cell markers (CD44, CD133, LGR5, ALDH, and pluripotency factors). It should be noted that bufalin’s ability to inhibit CD44 expression is consistent with previous studies [40,41,47]. CD44 is one of the most prominent markers and a key regulator of CSCs. CD44 knockdown can inhibit EMT and therefore inhibit the proliferation and invasion of CRC CSCs, as well as tumorigenesis and metastasis in vivo. Furthermore, CD44 + CRC cell populations have higher expression of the C-Kit receptor tyrosine kinase compared with CD44- cell populations [48]. Seimiya et al. found that CD44+ CRC CSCs can be divided into CD44 stable cells and CD44 transient cells, among which CD44 stable cells continuously and stably express CD44. Comparatively, the CD44 transient cells rapidly transform into CD44- cells. Additionally, CD44 stable cells express a higher level of C-Kit than CD44 transient cells, and knockout of C-Kit in CD44 stable cells attenuates CSC characteristics [49]. The Rothenberg team found that C-Kit can promote the CRC growth in vitro and in vivo, and revealed that CD44+/C-Kit+ cells were more tumorigenic than their CD44+/C-Kit- counterparts. Moreover, CD44+/C-Kit+ crypt epithelial cells promoted organoid formation from isolated LGR5+ crypt base cells [23,50]. However, they did not find a definitive association between C-Kit expression and clinical outcomes in patients. These results indicate that C-Kit plays an important role in the CRC CSC niche, and that further work should be performed to better understand the processes underlying its activity, especially in models of human CRC. 

Cancer stem cells and EMT processes are interrelated in the progression of CRC [51,52,53]. We initially began our study with a focus on CD44 and CD44+ CSCs in CRC. When treated with bufalin, all CRC lines showed downregulation of CD44 at a concentration of 40 nM, but other markers were more sensitive in their response, suggesting that they are upstream of CD44. Markers consistently altered at lower bufalin concentrations (20 nM) were C-Kit, the CSC marker ALDH1A1, and the EMT factor Slug, which has previously been shown to be a regulator of CD44+ CSCs in breast cancer [25]. Through network analysis, we found that C-Kit and Slug were known pathway interactors. Furthermore, analysis of human CRC RNA-Seq data showed that the expression of Slug and C-Kit mRNA was positively correlated, suggesting the potential for a C-Kit/Slug signaling axis that connects bufalin anti-stemness and EMT inhibitory effects in CRC. 

Studies in a variety of tumors have shown that C-Kit regulates EMT. For example, inhibition of C-Kit expression can significantly reduce cell proliferation, colony formation, invasion, and tumorigenesis in gefitinib-resistant NSCLC. Molecularly, this is accompanied by downregulation of CSC markers and pro-EMT proteins vimentin, N-cadherin, and Slug, and restoration of E-Cadherin [26]. Additionally, Slug is a transcriptional regulator of C-Kit in hematopoietic stem cells (HSCs), and the balance between Slug and C-Kit is important to maintain the regenerative potential of HSCs in vivo. In this context, C-Kit signaling enhances Slug expression, which inhibits C-Kit transcription and alters the self-renewal capacity of HSCs, suggesting a negative feedback loop mechanism [54]. In contrast to these findings, Procopio et al. found that multidrug-resistant malignant mesothelioma (MM) cells expressing C-Kit and its ligand simultaneously had higher Slug mRNA expression. Importantly, C-Kit or Slug knockdown was able to enhance the sensitivity of MM multidrug-resistant cells to chemotherapies, while overexpression of C-Kit in MM cells upregulated Slug and increased resistance to chemotherapy. MM cells expressing Slug also showed increased resistance to chemotherapy [27]. In the current study, we overexpressed C-Kit in the SW480 cell line, which has low endogenous C-Kit expression. Matrigel spheroid assays revealed that overexpression of C-Kit significantly increases spheroid formation capacity, demonstrating that C-Kit can promote CRC stemness. Additionally, C-Kit-overexpressing SW480 cells were significantly resistant to bufalin. The results of Western blot and qPCR showed that Slug expression increased significantly after C-Kit overexpression. Similarly, Slug overexpression in DLD1 cells resulted in a significant increase in the number of spheroids and resistance to bufalin treatment, while inducing C-Kit expression. Furthermore, Slug overexpression increased the expression of both CD44 and ALDH1A1. Our combined findings reveal a positive feedback loop mechanism between C-Kit and Slug in CRC, which may be of particular importance in CD44+ CSC-like populations. These results support previous findings of a potential C-Kit/Slug positive feedback loop in studies of human malignant mesothelioma and NSCLC [26,27], but are partly contrary to results in murine hematopoiesis and self-renewal [54]. 

One shortcoming of our current work is the dependence on C-Kit and Slug overexpression models to demonstrate the proposed positive feedback loop. It will be necessary to perform C-Kit/Slug knockdown or knockout experiments to further support the current hypothesis. Additionally, experiments with existing pharmacological C-Kit inhibitors may elucidate the relationship between C-Kit and Slug in CRC stemness. Finally, we note that the CpG island promoter region of the C-Kit gene contains a Slug binding motif/site (Hg38: Chr4:54657572-54657584), which may be manipulated in future studies to better understand the regulatory mechanisms that occur between C-Kit and Slug. In general, we conclude, based on current findings and previous studies, that C-Kit and Slug are closely related in human malignancies and that future studies focused on either target should take this into consideration due to their relevance to both the CSC and EMT processes.

In this study, we used PDOs to further explore the efficacy of bufalin against CRC and the role of C-Kit/Slug signaling pathway. Studies in CRC demonstrate that PDOs can accurately reproduce the characteristics of the tissue they come from, including the molecular, histological, and phenotypic factors. Importantly, they maintain realistic molecular and cellular heterogeneity, which is recognized as a key factor in CRC progression and therapy response. Multiple studies have shown that organoids can accurately reproduce the morphological structure and gene expression profile of CRC, including CMS classification [16], further suggesting that they are an effective ex vivo tool to study CRC heterogeneity. In general, PDOs can be used to predict prognosis and chemoresistance in CRC, and are suitable for high-throughput screening of candidate drugs [18]. Li et al. cultured PDOs from matched primary and liver metastatic tumor tissues of CRC patients, and found that liver metastatic PDOs had stronger tumorigenesis and metastasis potential linked to pluripotency factor SOX2 [55]. Further studies of metastasis-derived CRC PDOs showed that they can successfully predict the effect of irinotecan chemotherapy in patients, including whether combination with 5-FU would provide clinical benefit [56]. Toshimitsu et al. demonstrated the fidelity of PDOs for high-throughput screening revealing that bromodomain and extra-terminal (BET) inhibitors exerted variable targeting activities depending on whether they were given to normal epithelium-derived or CRC-derived organoids [57]. Another potential use for organoids is exemplified by the determination of chimeric antigen receptor-mediated cytotoxicity and therapeutic efficacy in CRC PDOs [58]. In this project, PDOs were established from endoscopic biopsy and used to observe the efficacy of bufalin treatment. Three out of five PDOs were sensitive to bufalin intervention. At the molecular level, bufalin negatively regulated the expression of C-Kit, Slug, CD133 and C-Myc and positively regulated the expression of the epithelial cell marker E-cadherin in these sensitive PDOs. In PDOs with weak or no response to bufalin (1208, 1101), C-Kit and Slug expression were not significantly down-regulated. Notably, these resistant PDOs also expressed relatively higher, but clinically non-significant levels of the serum markers AFP and CA19-9 (Table 1). 

## 4. Materials and Methods

### 4.1. Establishment and Cultivation of PDOs

The study was carried out according to the Declaration of Helsinki and the CIOMS International Ethics Guidelines for Human Biomedical Research, and approved by the Ethics Committee of Fuzhou Traditional Chinese Medicine Hospital, AF/SC-08/03.3, approval date: 15 April 2021. All patients who contributed tissue for this study provided their informed consent. To establish PDOs, tissues from patients with colorectal cancer were obtained during colonoscopy and washed thoroughly with 5 mL of cold D-PBS (#37350, STEMCELL, Vancouver, BC, CA) containing Primocin (#ant-pm-2, InvivoGen, Carlsbad, CA, USA) at least 3 times. Excess D-PBS was discarded, leaving 1 mL total volume which was then transferred to a sterile 1.5 mL microcentrifuge tube. The tissue fragments were allowed to settle by gravity, and the liquid was discarded. The tissues were then cut into fragments of about 5 mm using sterile surgical scissors. Next, 1 mL of Gentle Cell Dissociation Reagent (GCDR; #7174, STEMCELL, Vancouver, BC, CA) was used to resuspend the tissue, and then transferred to a new 15 mL centrifuge tube. The 1.5 mL centrifuge tube was washed with 1 mL of GCDR to capture any fragments and transferred to the same 15 mL centrifuge tube. GCDR was added to the 15 mL tube at a total volume of 10 mL, placed on a shaker for 10 min, and then incubated for 1 h at 37 °C. The dissociated crypts were centrifuged at 290× *g* for 5 min and the supernatant was discarded. The remaining pellet was vigorously pipetted up and down with 1 mL of precooled DMEM/F12 (#36254, STEMCELL, Vancouver, BC, CA) + 1% BSA (#A8010, Solarbio, Beijing, CN) solution 20 times. The resulting fluid was transferred to a 15 mL centrifuge tube through a 70 µm cell strainer (#27216, STEMCELL, Vancouver, BC, CA) for filtration and the above washing and filtration steps were repeated once again. After filtration, the crypts were counted under a microscope. The solution was then centrifuged at 200× *g* for 5 min, and a 1:1 mixture of crypt suspension and reduced growth factor matrigel (#356231, Corning, Corning, NY, USA) was seeded at 50 µL/well with 1000 crypts per well to a preheated 24-well plate and placed in the incubator for 30 min until the matrigel solidified. Finally, 750 µL of Complete IntestiCult Organoid Growth Medium (#6005, STEMCELL, Vancouver, BC, CA) was added to each well and incubated at 5% CO_2_ and 37 °C. During establishment, the maturation of organoids was observed daily and pictures were taken at appropriate intervals. Organoids were expanded and then cryopreserved for future studies according to the protocol for CS10 (#7931, STEMCELL, Vancouver, BC, CA) 

### 4.2. PDO Western Blot Assay

The PDOs were prepared in a 24-well plate as described above. After one week of culture, bufalin (#HY-N0877, MCE, Monmouth Junction, NJ, USA) (40 nM) or DMSO (#D2650, Sigma, St. Louis, MO, USA) was added to the growth medium for 3 days. The medium was then discarded and the wells were washed with D-PBS 3 times. Next, a 1 mL tip was used to break the matrigel which was then resuspended vigorously, transferred to a 15 mL tube, and centrifuge at 200× *g* for 5 min. Following centrifugation, the supernatant was discarded and the remaining organoids were lysed with a mixture of MPER (#78501, Thermo-Fisher, Waltham, MA, USA), PMSF (#KGP610, Keygen, Nanjing, Jiangsu, CN) and protease inhibitor cocktail (#HY-K0010, MCE, Monmouth Junction, NJ, USA). The rest of the procedure was performed as described in the standard Western blot protocol. 

### 4.3. PDO Growth Assay

To assess growth, 100 crypts/well were seeded into a 96-well plate, and cultured for about a week until mature organoids formed. Bufalin was then added at concentrations of 20 and 40 nM for treatment and growth was observed daily. Organoid counts and photographs were taken every other day for up to 10 days depending on the individual organoid response. At the end of the experiment, Calcein-AM (#KGMP012-1, Keygen, Nanjing, Jiangsu, CN) was added to visualize the remaining live organoids. After 1 h, fluorescence photos were taken at 5× and 20× magnifications. ImageJ (Bethesda, MD, USA) was used to measure the size of the organoids.

### 4.4. Cell Lines and Culture

CRC cell lines (HCT116, DLD1, SW480, and HCT15) were purchased from the Cell Bank of the Typical Culture Preservation Commission, Chinese Academy of Sciences (Shanghai, CN), where they were verified by short tandem repeat analysis and confirmed mycoplasma-free by PCR. Cells were cultured in high-glucose medium (#SH30022.01B, Hyclone, Logan, UT, USA) containing 10% FBS (#ST30-3302, PAN, Aidenbach, Bavaria, Germany) and 1% antibiotic-antimycotic (#2108964, Gibco, Grand Island, NY, USA) at 5% CO_2_ and 37 °C. 

### 4.5. Cell Proliferation and LIVE/DEAD Assays

HCT116, DLD1, or SW480 cells were seeded in 96-well plates at 10^5^ cells per well. When reaching 60%–70% confluence, cells were treated with bufalin at various concentrations (0–10 μM) for 72 h at 37 °C. Afterwards, 7 µL MTT (#M1025, Solarbio, Beijing, CN) was added to each well and incubation continued until punctate blue-purple crystals appeared. DMSO containing 1 mM ammonium hydroxide (#30501, Sigma, St. Louis, MO, USA) was then added to dissolve the crystals and the absorbance values were measured at 492 nm on a microplate reader (Spark Multimode Microplate Reader, TECAN, Männedorf, CH). For LIVE/DEAD assay, HCT116, DLD1 and SW480 cells were seeded into 96-well plates at 10^4^ cells/well and cultured at 37 °C in a 5% CO2 incubator. When cells reached 60–70% confluence, bufalin was added at a concentration of 12.5, 25, 50, or 200 nM and DMSO was used as vehicle control. Triplicate wells were treated in each group, and the cell status was observed daily. At 48 h post-treatment, Calcein-AM (LIVE) with a final concentration of 5 µM and propidium iodide (#KGA107, Keygen, Nanjing, Jiangsu, CN) (PI/DEAD) dye with a final concentration of 1 µg/mL were added to each well and incubated for 30 min. Finally, the fluorescence intensity was detected using a microplate reader and fluorescent microscope.

### 4.6. Matrigel Spheroid-Forming Assay 

For each well of a 96-well plate, a 25 µL cell suspension (containing 500 cells) was mixed with 25 µL matrigel and allowed to solidify in an incubator for 30 min. After the matrigel solidified, 100 µL of DMEM medium containing 0.5% FBS was added on top of the well. Varying concentrations of bufalin (8 nM, 20 nM, 40 nM) were added the next day and the medium was changed every 3 days. After 10 days of culture, the medium was removed from each well, followed by a standard formalin fixation procedure. The spheroids were counted and photographed and the spheroid size was quantified using ImageJ.

### 4.7. Transwell Migration and Invasion Assay 

For the transwell migration assay, a 2 × 10^5^ cell/mL suspension (HCT116, DLD1, or SW480) in serum-free medium and 200 µL was added to the upper chamber of the transwell. Afterwards, 700 µL of 10% FBS-containing medium was added below the transwell to act as a chemoattractant. Bufalin (40 nM) was immediately added to the upper chamber. After 48 h of incubation, the medium was removed and the transwell was fixed in 10% neutral buffered formalin (#G2161, Solarbio, Beijing, CN) for 10 min. The cells attached to the layer inside the upper chamber were carefully wiped with cotton swabs, and 0.1% crystal violet solution (#G1062, Solarbio, Beijing, CN) was added to both the upper chamber and bottom well for 15 min. Crystal violet was then removed and excess stain was washed away with tap water. The migrated cells were counted and photographed at a 10× magnification using a microscope after drying. The transwell invasion assay was performed according to the same procedure using a BD BioCoat Matrigel Invasion Chamber (#354480, Corning, Corning, NY, USA).

### 4.8. Plasmid Transfection 

Human C-Kit (NM_000222)-pcDNA3.1(+)-EGFP-N plasmid (KIT-OE) and EGFP vector control plasmids were constructed by Hunan Feng Hui Company (Changsha, Hunan, CN), and confirmed by Sanger sequencing, qPCR, and Western blotting. For transfection, SW480 cells were seeded in a 6-well plate and transfected with KIT-OE or EGFP after reaching 70% confluency using Lipofectamine 3000 (#L3000008, Thermo Fisher, Waltham, MA, USA), according to the manufacturer’s instructions (#L3000150, Invitrogen, Waltham, MA, USA). After 48 h transfection, cells were collected for downstream assays. Fluorescent microscopy was used to confirm the transfection efficiency.

### 4.9. Lentivirus Infection 

Cells were seeded in a 6-well plate and infected with lentivirus when they reached 50% confluence. Lentiviral particles used included Slug lentiviral activation particles (#sc-400389-LAC), C-Kit lentiviral activation particles (#sc-400106-LAC), copGFP control lentiviral particles (#sc-108084), and control lentiviral activation particles (#sc-437282) (Santa Cruz Biotechnology, Dallas, TX, USA). Infected clones were selected by 2 µg/mL puromycin dihydrochloride (#sc-108071, Santa Cruz Biotechnology, Dallas, TX, USA). After selecting antibiotic-resistant clones for one week, western blot was used to confirm protein expression and copGFP controls were used to confirm stable selection. Stable cell lines were expanded, cryopreserved, and used for downstream analysis.

### 4.10. ALDEFLUOR Assay 

Cells were pretreated with bufalin (40 nM) or DMSO for 24 h (HCT116) or 48 h (DLD1). Cells were collected and suspended in 1 mL of ALDEFLUOR assay buffer at 2 × 10^5^ cells/mL. The following steps were performed according to the manufacturer’s instructions (#01700, STEMCELL, Vancouver, BC, CA). Flow cytometry measurements were taken using a BD FACSCalibur apparatus (Franklin Lakes, NJ, USA) and analyses were performed using FlowJo 10.6.2 (BD, Franklin Lakes, NJ, USA).

### 4.11. Separation of Nuclear and Cytoplasmic Proteins 

Cells were pretreated with bufalin (40 nM) or DMSO for 24 h (HCT116) or 48 h (DLD1, SW480, and HCT15). Afterwards, cells were collected by centrifugation and the resulting cell pellets were washed with PBS. 1 × 10^7^ cells were counted, transferred to a microcentrifuge tube, and pelleted by centrifugation at 500× *g* for 3 min. After centrifugation, the supernatant was removed, leaving the cell pellet as dry as possible. The ice cold CER I was then added to the cell pellet following the Ne-per Nuclear and Cytoplasmic Extraction Kit protocol (#78833, Thermo-Fisher, Waltham, MA, USA). The resulting cytoplasmic and nuclear extracts were denatured using SDS, and then used for western blot.

### 4.12. Immunofluorescence Microscopy 

For each treatment, a 300 µL cell suspension (10^5^ cells/mL) was seeded in an Eight-well Glass Chamber Slide (#80806, Ibidi, Grafelfing, Germany). Bufalin (40 nM) was added the next day and the medium was discarded after a specific time point (24 h for HCT116 and DLD1, and 48 h for SW480 and HCT15). The slides were then washed with cold PBS 3 times and fixed with neutral-buffered formalin for 10 min. The cell membrane was then permeabilized with 0.1% Triton-X (#KGF011, Keygen, Nanjing, Jiangsu, CN) for 10 min, washed with cold PBS 3 times and incubated with β-catenin primary antibody (aAb32672, Abcam, Cambridge, UK) diluted in 10% donkey serum (#SL050, Solarbio, Beijing, CN) overnight at 4 °C. The following day, the primary antibody was removed and the cells were washed with cold PBS 3 times. FITC-conjugated secondary antibody (donkey anti-rabbit IgG; #ab150073, Abcam, Cambridge, UK) diluted in 10% donkey serum was then added and incubated in darkness for 1 h. Next, the wells were washed 3 times with cold PBS and DAPI counterstaining was performed according to manufacturer instructions (#C1005, Beyotime, Shanghai, CN). Photos were taken at a magnification of 40x with a Leica DMI4000 B fluorescent microscope (Wetzlar, Germany). DAPI and FITC images were merged using ImageJ.

### 4.13. Western Blot 

After treatment with bufalin (20 or 40 nM) or DMSO for a specific period of time (24 h for HCT116, 48 h for DLD1 and SW480), the cells were washed with PBS and lysed with a mixture of MPER (#78501, Pierce, Rockland, IL, USA), PMSF, and protease inhibitor cocktail. The concentration of total protein was determined by BCA assay (#23227, Thermo-Fisher, Waltham, MA, USA) and the proteins were separated by SDS-PAGE and transferred to a PVDF membrane. After blocking with 1× blocking buffer (#ab126587, Abcam, Cambridge, UK) for 2 h at RT, the membrane was incubated with primary antibody overnight at 4 °C on a rocking platform. The antibodies used included β-actin (#SC-47778, Santa Cruz Biotechnology, Dallas, TX, USA), GAPDH (#60004-1-IG, Proteintech, Chicago, IL, USA), N-cadherin (D4R1H) XP^®^ (#13116, CST, Danvers, MA, USA), ZEB1 (#3396, CST, Danvers, MA, USA), Vimentin (D21H3) XP^®^ (#5741, CST, Danvers, MA, USA), CD44 (#60224-1-LG, Proteintech, Chicago, IL, USA), C-Myc (#10828-1-AP, Proteintech, Chicago, IL, USA), NANOG (#67255-1-lg, Proteintech, Chicago, IL, USA), SOX2 (#66411-1-lg, Proteintech, Chicago, IL, USA), CD133 (#66666-1-lg, Proteintech, Chicago, IL, USA), C-Kit (#18696-1-AP, Proteintech, Chicago, IL, USA), LGR5 (#bs-20747R, Bioss, Beijing, CN), KLF4 (#bs-1064R, Bioss, Beijing, CN), ALDH1A1 (#K000356P, Solarbio, Beijing, CN), and Slug (#A1057, ABclonal, Wuhan, CN). After incubation with primary antibody, the membrane was washed with TBST 3 times for 5 min each time, and the secondary antibody (HRP-conjugated Affinipure Goat Anti-Mouse IgG(H + L): #SA00001-1 or HRP-conjugated Affinipure Goat Anti-Rabbit IgG(H + L): #SA00001-2, Proteintech, Chicago, IL, USA) was incubated for 2 h at RT. After removal of the secondary antibody, the membranes were washed with TBST 3 times for 5 min each time. SuperLumia ECL Plus HRP Substrate (#K22030, Abbkine, Wuhan, CN) was used for chemiluminescence, and the blot images were obtained using a BioRad Gel Doc XR+ system (Hercules, CA, USA). Gray values were evaluated using ImageJ gel quantification tools. Raw Western blot images are available in Figures S3–S35.

### 4.14. Bioinformatics and Statistical Analysis 

Statistical analysis was performed using GraphPad Prism v8 (GraphPad Software, San Diego, CA, USA). For relative comparisons, the Student’s T-test was used to detect significant differences in mean between groups. For time and dose-dependent analyses, ANOVA was used, where appropriate. For growth inhibition curves, a sigmoidal-dose response curve was fit and an IC_50_ value was calculated. For network analysis, genes of interest were surveyed using the GeneMANIA algorithm [59]. For bioinformatic analysis of CRCs, the TCGA colorectal adenocarcinoma (COADREAD) RNA-Seq dataset (Illumina HiSeqV2) was downloaded from the UCSC Cancer Genome Browser (xenabrowser.net) on 22 May 2020. The correlation analysis was performed in R v3.2 using the Pearson method.

## 5. Conclusions

In conclusion, our findings demonstrated that C-Kit and Slug mediate the stemness of CRC through a mutually regulated mechanism of action. Additionally, we found that bufalin can target the C-Kit/Slug signaling axis to dampen or reverse this phenomenon, leading to significant reductions in CSC marker expression, invasive/EMT characteristics, and functional stemness. Finally, we demonstrated the inhibition of growth following bufalin treatment in organoids derived from CRC patients, which show individualized responses consistent with a C-Kit/Slug-dependent effect. Overall, further studies are warranted to better understand C-Kit/Slug signaling in CRC tumorigenesis and progression and may lead to new therapeutic avenues for targeting CRC CSCs.

## Figures and Tables

**Table 1:** Demographic and clinical characteristics of patients undergoing endoscopic biopsy.

Sample	T0519	T0528	T0714	T1208	T1101
Gender	M	M	M	F	M
Age	80	84	73	72	81
Pathology	Villous tubular adenoma	Adenocarcinoma	Adenocarcinoma	Adenocarcinoma	Adenocarcinoma
Anatomic site	Rectum	Sigmoid Colon	Rectum	Rectum	Rectum
AFP (ng/mL)	1.6	1.34	N/A	2.37	2.82
CA-125 (U/mL)	10.8	6	N/A	12.74	N/A
CEA (ng/mL)	2.56	1.77	N/A	1.58	7.71
CA19-9 (U/mL)	<2	2.1	N/A	6.5	18.31

## Data Availability

The Cancer Genome Atlas Colon Adenocarcinoma dataset is available for download at xenabrowser.net and maintained by the United States National Institutes of Health at gdc.cancer.gov. All other experimental data used in the study are available from the corresponding authors by reasonable request.

## Supplementary Materials

The supporting information can be downloaded at: https://www.mdpi.com/article/10.3390/ijms232113354/s1.
