# Supplementary material for: Bufalin Inhibits Tumorigenesis, Stemness, and Epithelial–Mesenchymal Transition in Colorectal Cancer through a C-Kit/Slug Signaling Axis
Source: Int J Mol Sci. 2022 Nov 1;23(21):13354. doi: 10.3390/ijms232113354 (PMC9656328; doi:10.3390/ijms232113354)
Supplement: Supplementary file 1 [file ijms-23-13354-s001.zip › Ding_et_al_S1-S2.pdf]

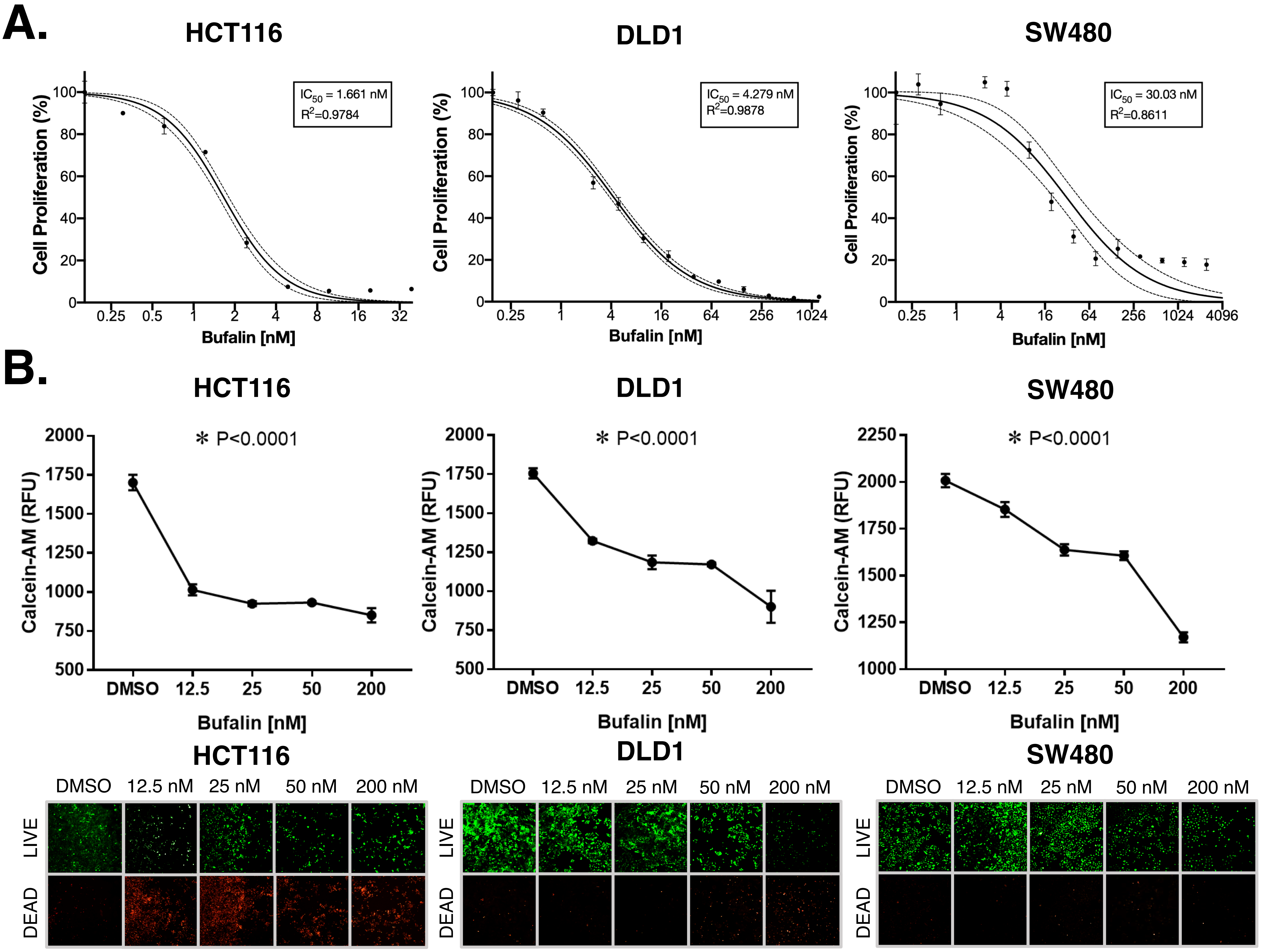

Figure S1. Bufalin inhibits the growth and viability of CRC cells. A. MTT assay results demonstrating that bufalin has a dose-dependent inhibitory effect on the proliferation of HCT116, DLD1 and SW480 after 72 h of treatment, with IC50 values ranging from 1.661 to 30.03 nM. **B.** Calcein AM (LIVE) and PI (DEAD) staining showing a significant decrease in living CRC cells after 48 h of bufalin treatment ranging from 12.5-200 nM concentrations.

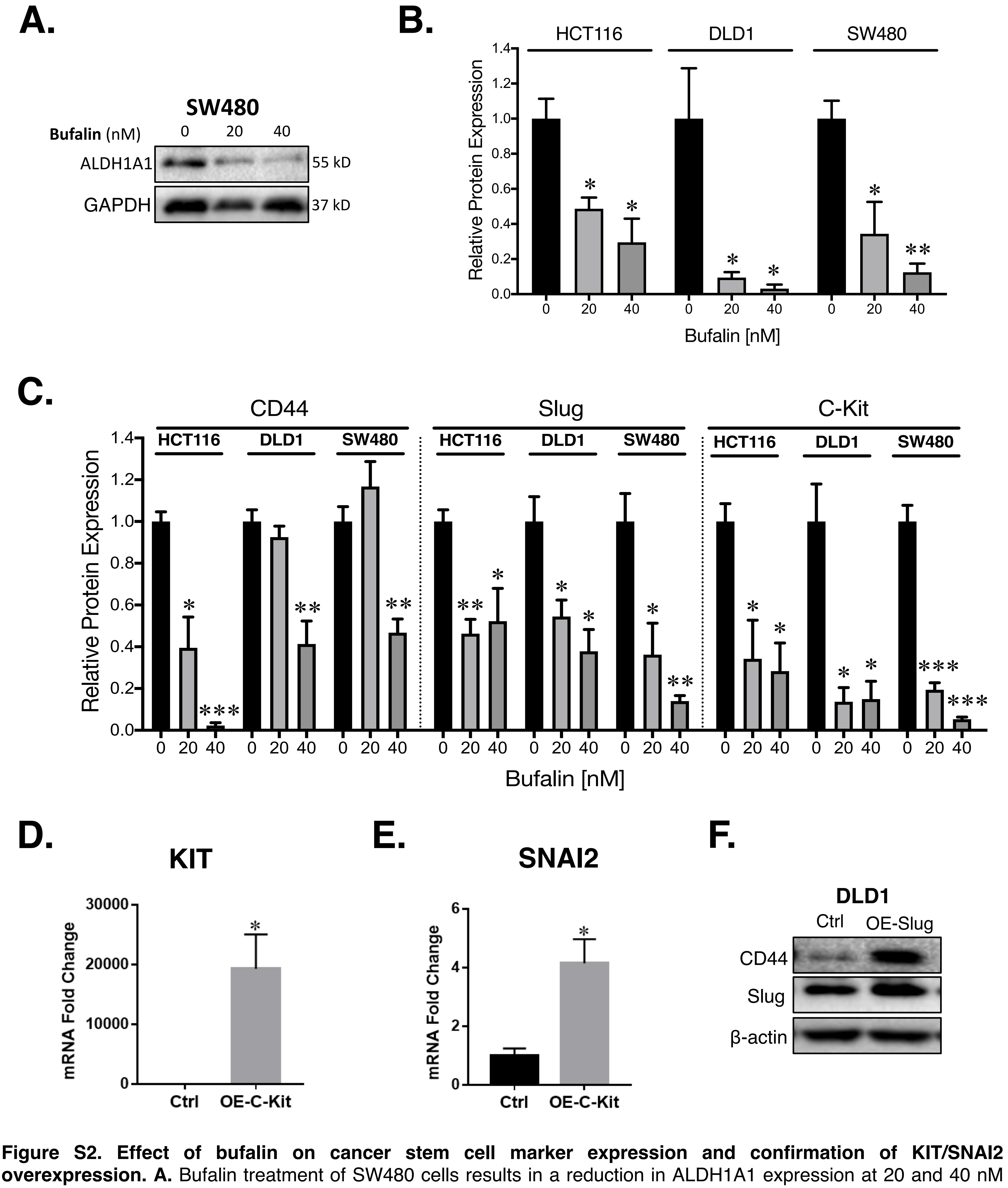

Figure S2. Effect of bufalin on cancer stem cell marker expression and confirmation of KIT/SNAI2 overexpression. A. Bufalin treatment of SW480 cells results in a reduction in ALDH1A1 expression at 20 and 40 nM doses. **B.** Quantification of ALDH1A1 after bufalin treatment in HCT116, DLD1, and SW480 cell lines revealing statistically significant decreases at 20 and 40 nM in all lines. **C.** Quantification of CD44, Slug, and C-Kit expression levels in HCT116, DLD1, and SW480 cell lines revealing variable decreases in expression for CD44 in SW480, and significant decreases for Slug and C-Kit at 20 and 40 nM in all cell lines. **D.** Confirmation of C-Kit mRNA overexpression in SW480 cells by qPCR. **E.** qPCR results for SNAI2 (Slug) demonstrating a significant 4-fold increase after C-Kit overexpression in SW480 cells. **F.** Overexpression of Slug in DLD1 cells results in strong upregulation of CSC marker CD44. (*p<0.05, **p<0.01)
