# Supplementary material for: Bufalin Inhibits Tumorigenesis, Stemness, and Epithelial–Mesenchymal Transition in Colorectal Cancer through a C-Kit/Slug Signaling Axis
Source: Int J Mol Sci. 2022 Nov 1;23(21):13354. doi: 10.3390/ijms232113354 (PMC9656328; doi:10.3390/ijms232113354)
Supplement: Supplementary file 1 [file ijms-23-13354-s001.zip › Ding_et_al_S3-S35.pptx]

## Slide 1
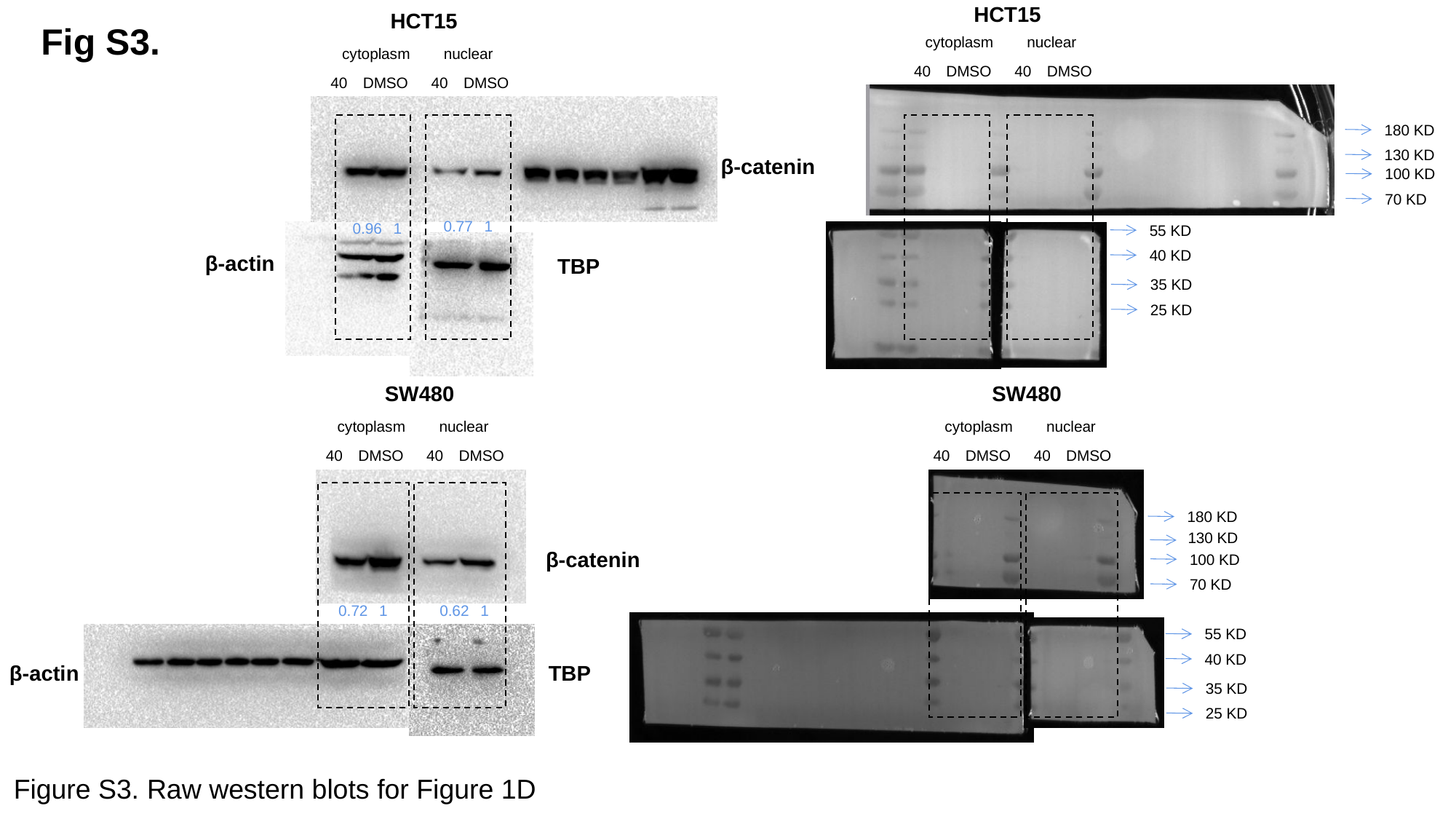

HCT15
HCT15
cytoplasm
nuclear
cytoplasm
nuclear
40
DMSO
40
DMSO
40
DMSO
40
DMSO
180 KD
130 KD
β-catenin
100 KD
70 KD
0.77
1
0.96
1
55 KD
40 KD
β-actin
TBP
35 KD
25 KD
Fig S3.
SW480
SW480
cytoplasm
nuclear
cytoplasm
nuclear
40
DMSO
40
DMSO
40
DMSO
40
DMSO
180 KD
130 KD
β-catenin
100 KD
70 KD
0.72
1
0.62
1
55 KD
40 KD
TBP
β-actin
35 KD
25 KD
Figure S3. Raw western blots for Figure 1D

## Slide 2
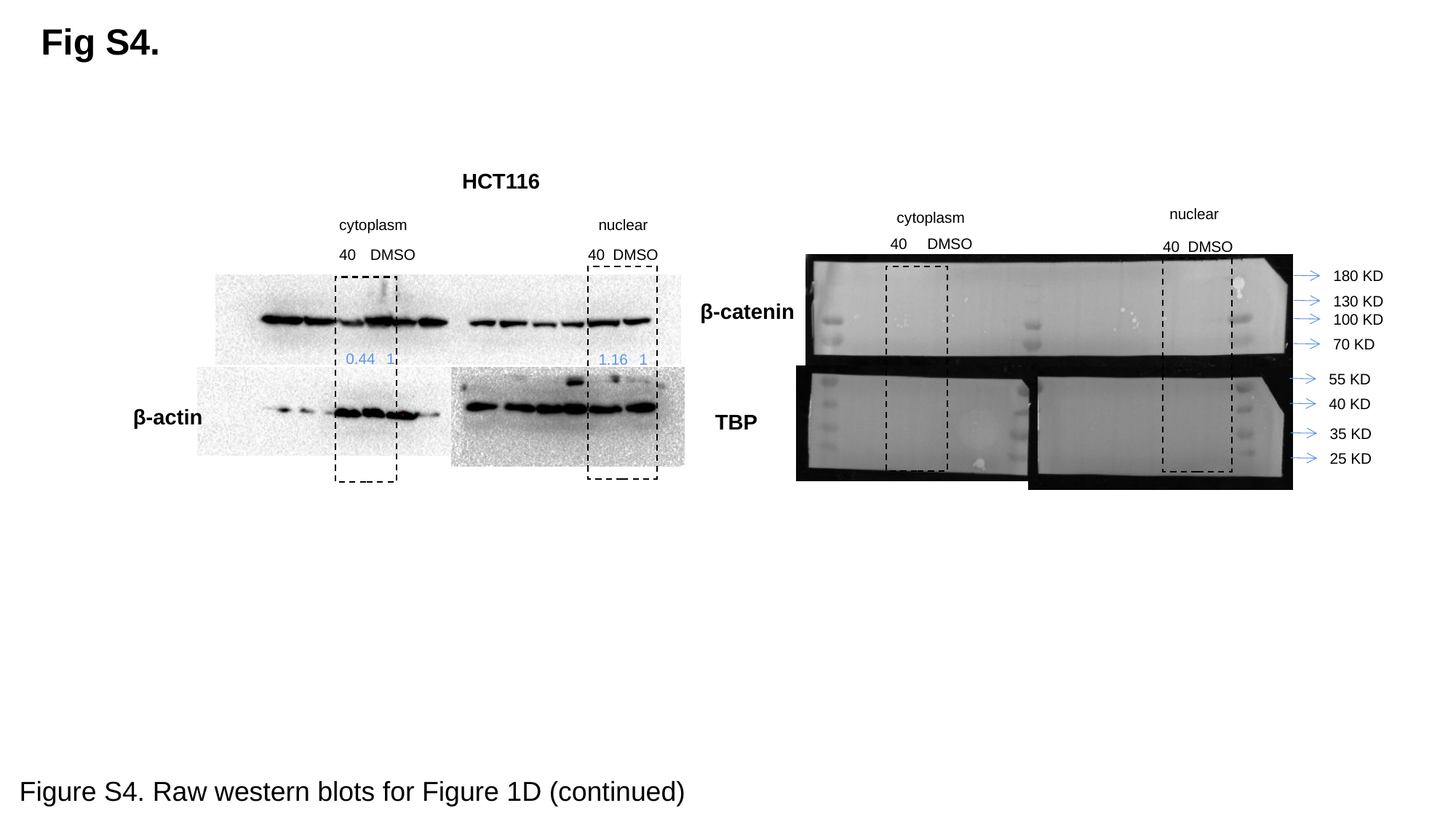

Fig S4.
HCT116
nuclear
cytoplasm
cytoplasm
nuclear
40
DMSO
40
DMSO
40
DMSO
40
DMSO
180 KD
130 KD
β-catenin
100 KD
70 KD
0.44
1
1.16
1
55 KD
40 KD
β-actin
TBP
35 KD
25 KD
Figure S4. Raw western blots for Figure 1D (continued)

## Slide 3
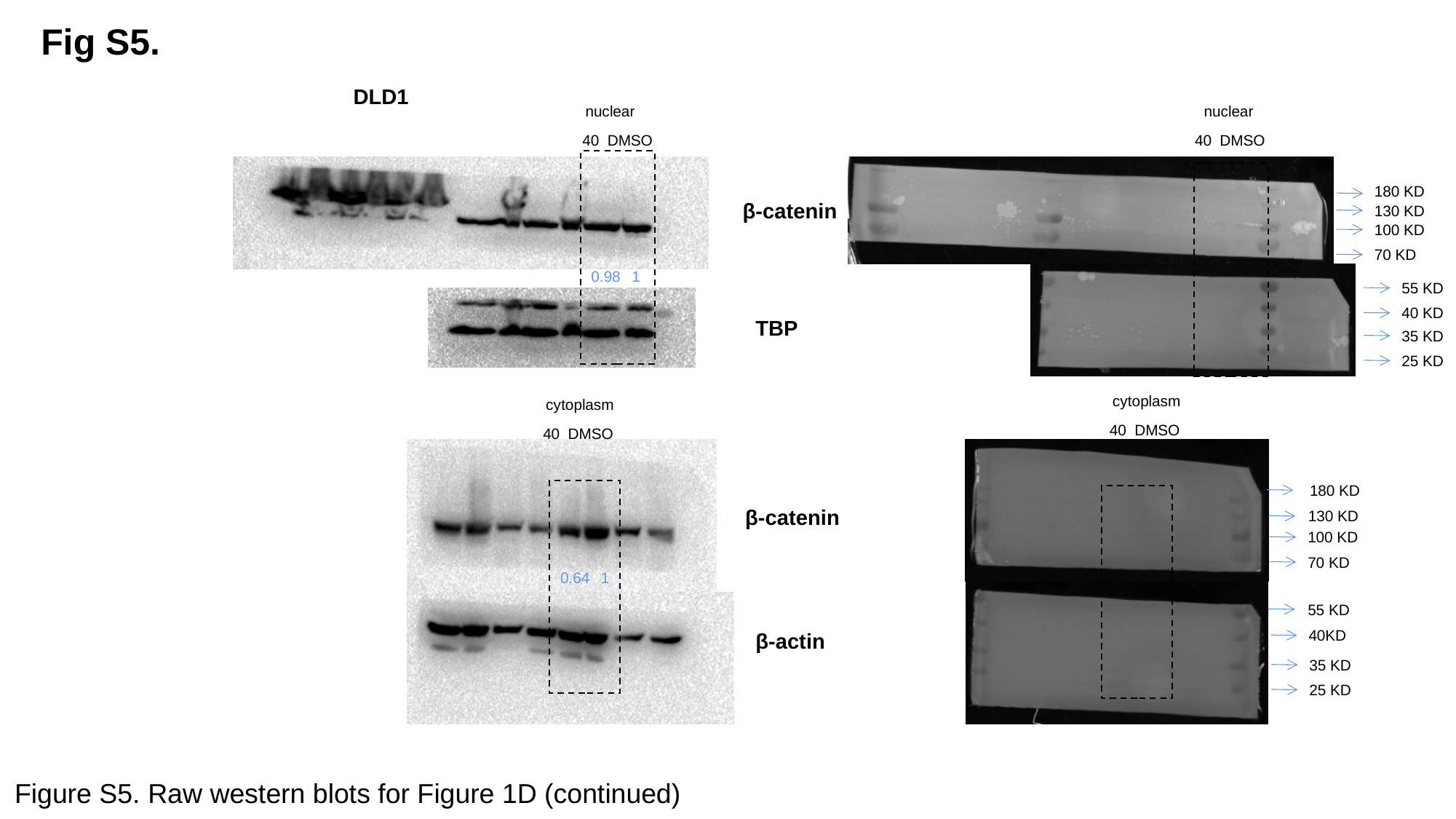

Fig S5.
DLD1
nuclear
nuclear
40
DMSO
40
DMSO
180 KD
β-catenin
130 KD
100 KD
70 KD
0.98
1
55 KD
40 KD
TBP
35 KD
25 KD
cytoplasm
cytoplasm
40
DMSO
40
DMSO
180 KD
β-catenin
130 KD
100 KD
70 KD
0.64
1
55 KD
40KD
β-actin
35 KD
25 KD
Figure S5. Raw western blots for Figure 1D (continued)

## Slide 4
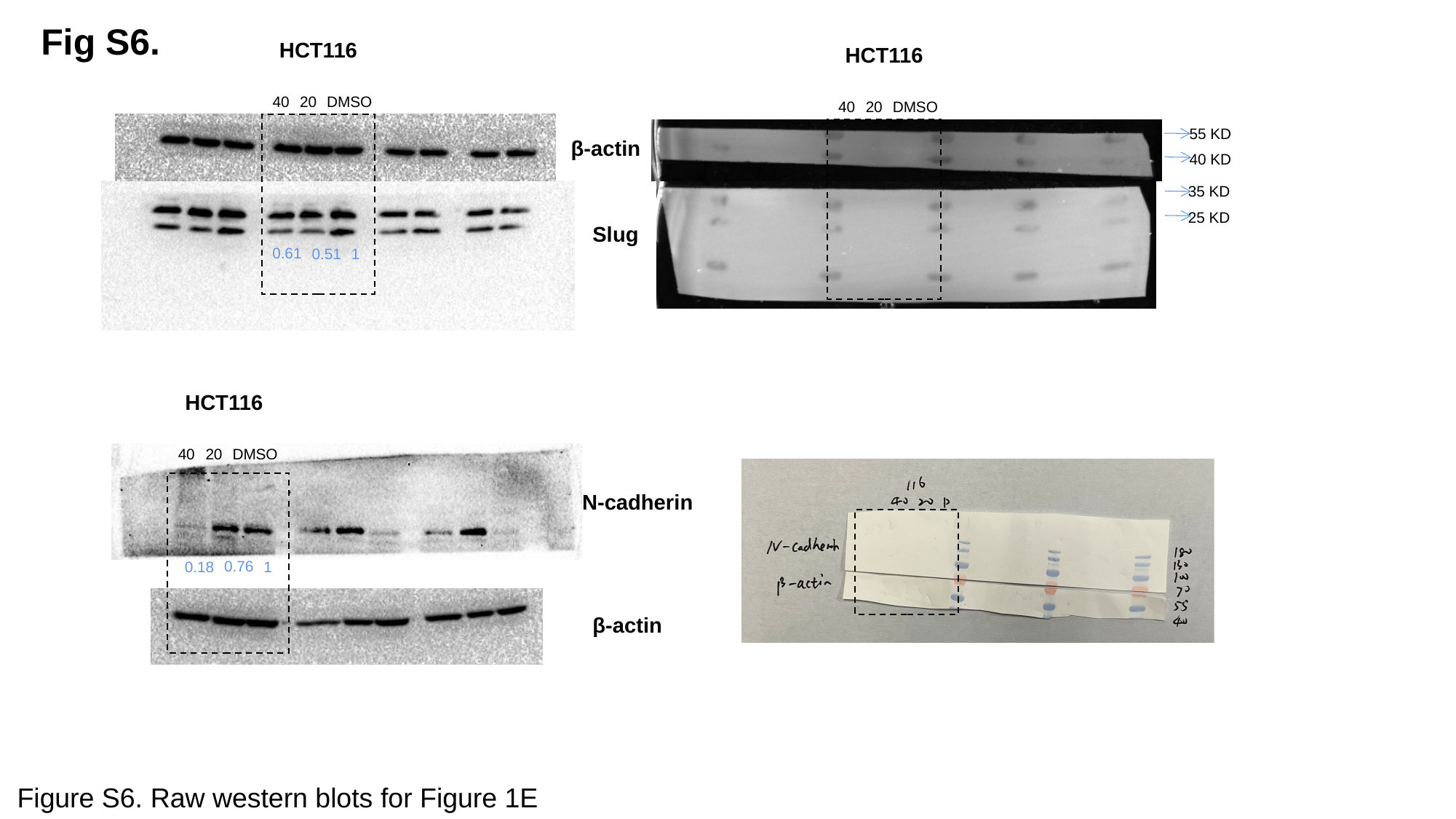

Fig S6.
HCT116
HCT116
40
20
DMSO
40
20
DMSO
55 KD
β-actin
40 KD
35 KD
25 KD
Slug
0.61
0.51
1
HCT116
40
20
DMSO
N-cadherin
0.76
1
0.18
β-actin
Figure S6. Raw western blots for Figure 1E

## Slide 5
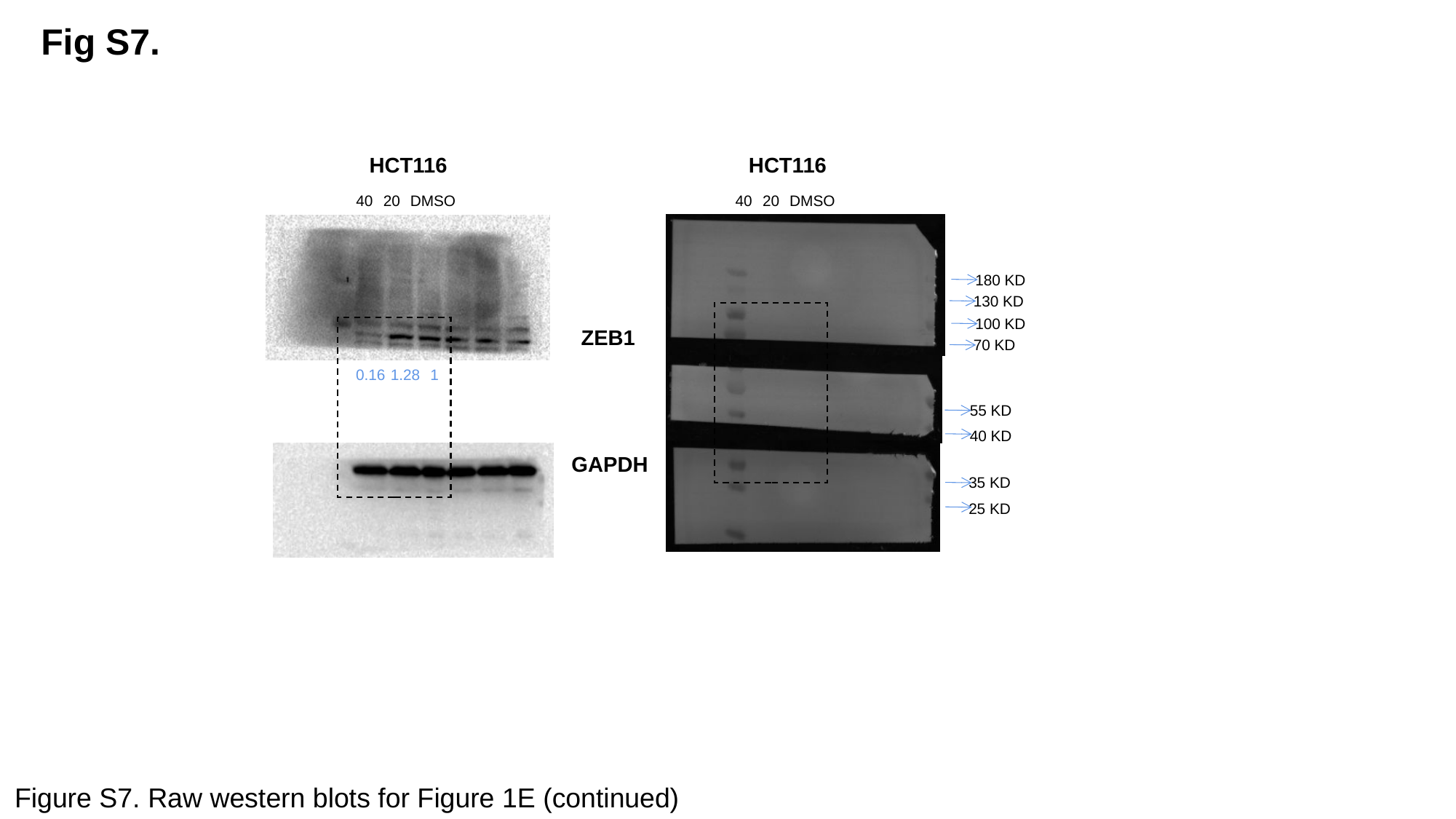

Fig S7.
HCT116
HCT116
40
20
DMSO
40
20
DMSO
180 KD
130 KD
100 KD
ZEB1
70 KD
1.28
0.16
1
55 KD
40 KD
GAPDH
35 KD
25 KD
Figure S7. Raw western blots for Figure 1E (continued)

## Slide 6
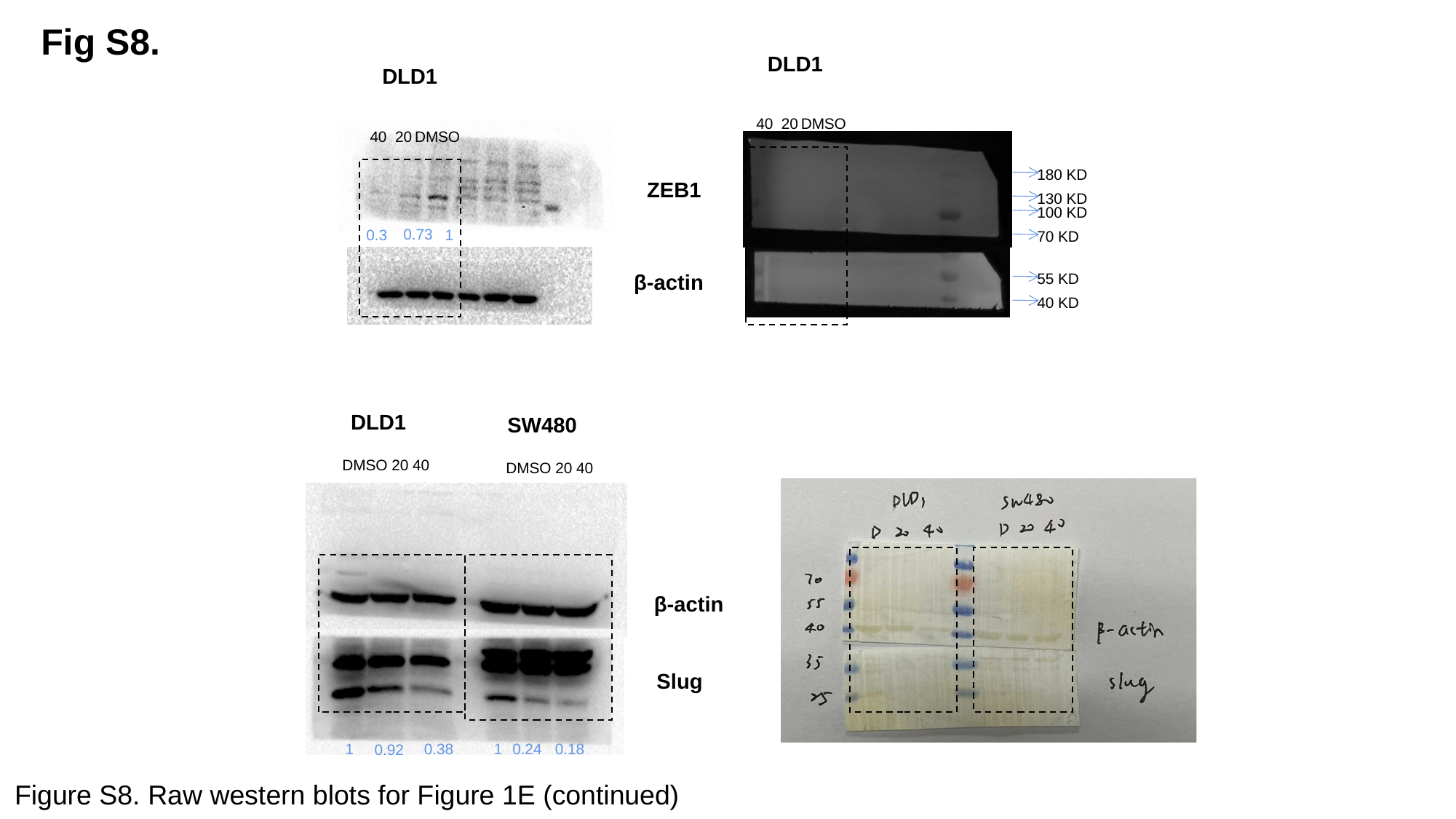

Fig S8.
DLD1
DLD1
40
20
DMSO
40
20
DMSO
180 KD
ZEB1
130 KD
100 KD
0.73
1
0.3
70 KD
β-actin
55 KD
40 KD
DLD1
SW480
DMSO 20 40
DMSO 20 40
β-actin
Slug
0.24
0.18
1
0.38
1
0.92
Figure S8. Raw western blots for Figure 1E (continued)

## Slide 7
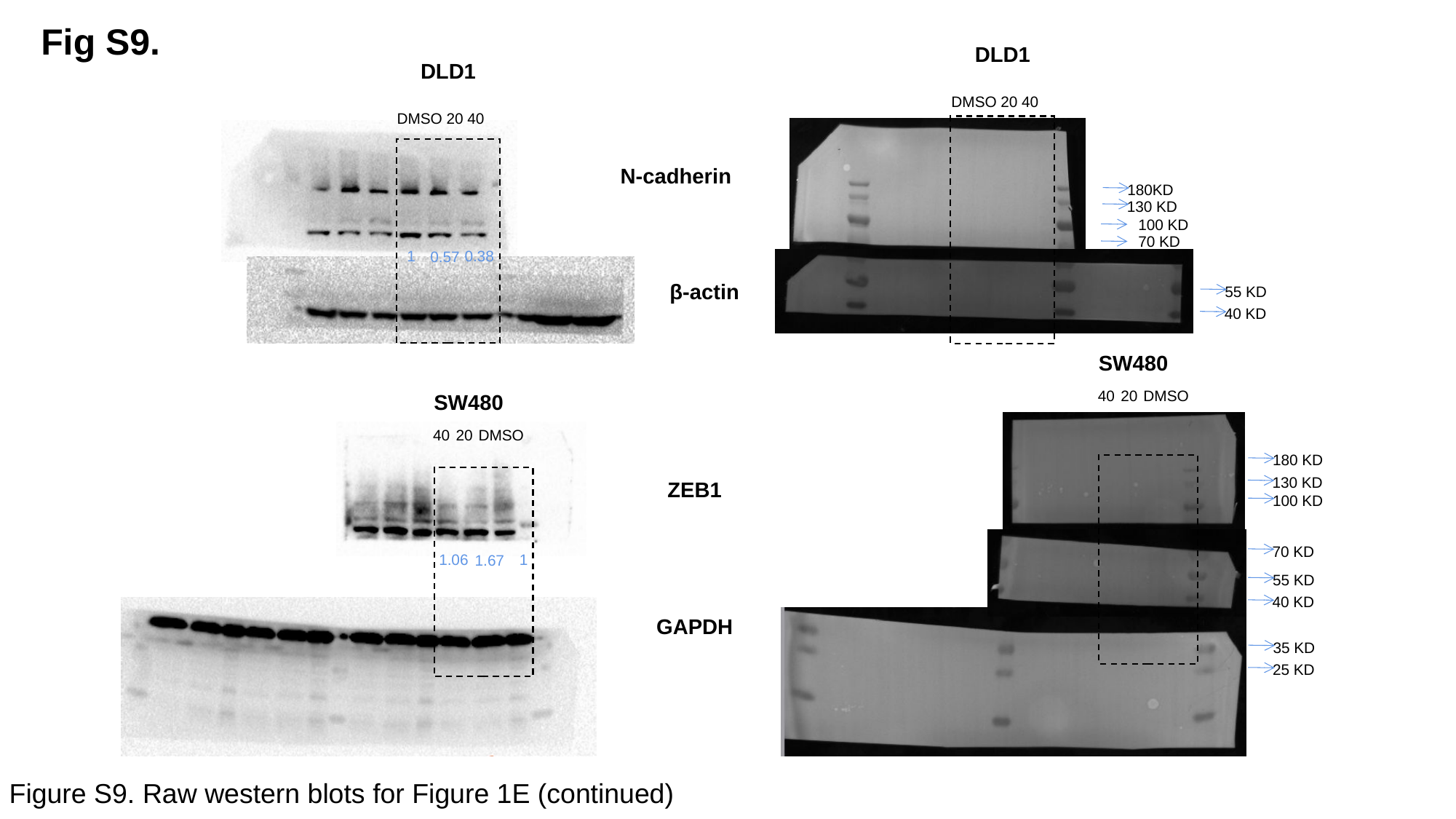

Fig S9.
DLD1
DLD1
DMSO 20 40
DMSO 20 40
N-cadherin
180KD
130 KD
100 KD
70 KD
0.38
1
0.57
β-actin
55 KD
40 KD
SW480
40
20
DMSO
SW480
40
20
DMSO
ZEB1
1.06
1
1.67
GAPDH
180 KD
130 KD
100 KD
70 KD
55 KD
40 KD
35 KD
25 KD
Figure S9. Raw western blots for Figure 1E (continued)

## Slide 8
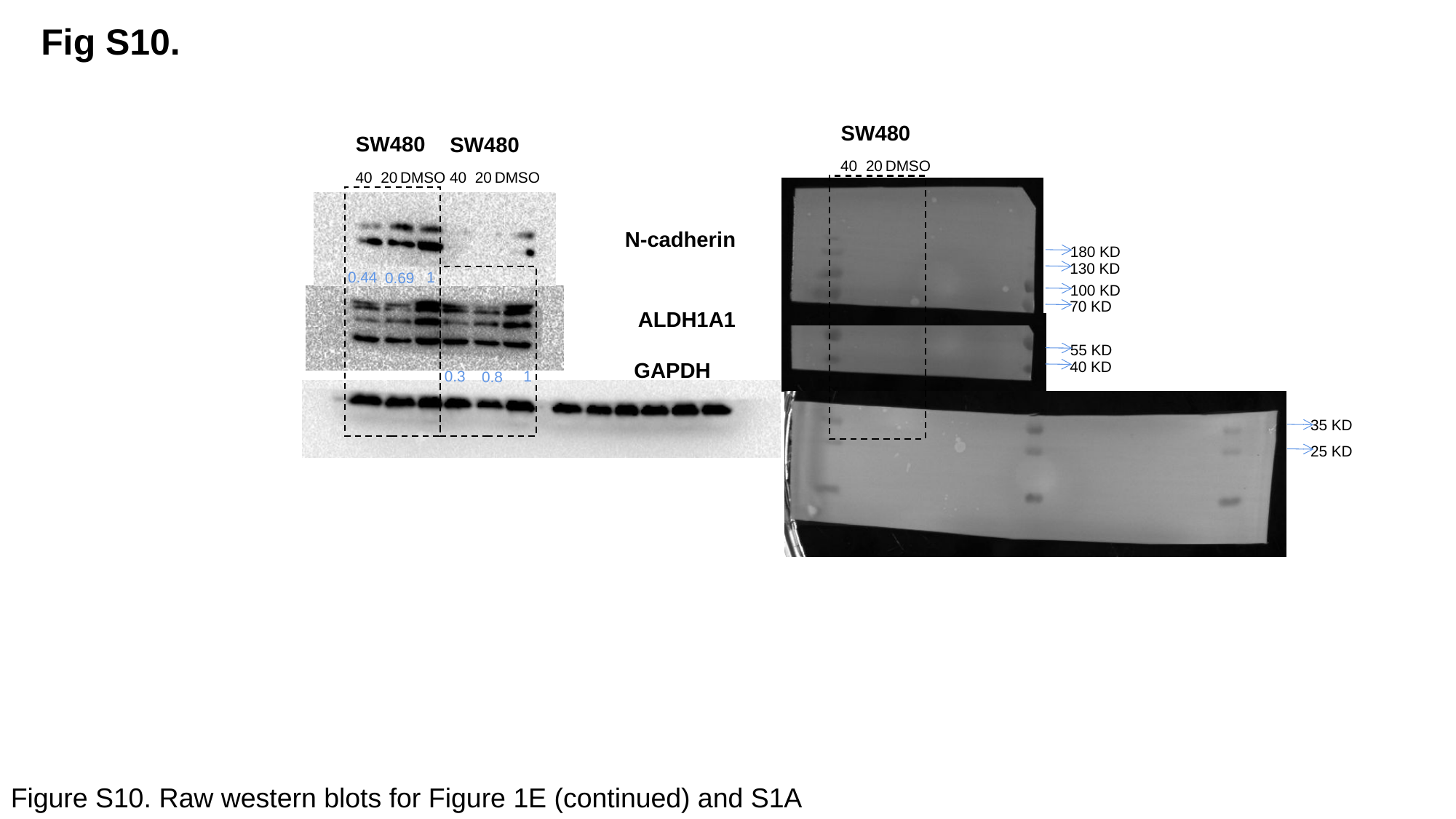

Fig S10.
SW480
SW480
SW480
40
20
DMSO
40
20
DMSO
40
20
DMSO
N-cadherin
180 KD
130 KD
1
0.44
0.69
100 KD
70 KD
ALDH1A1
55 KD
GAPDH
40 KD
1
0.3
0.8
35 KD
25 KD
Figure S10. Raw western blots for Figure 1E (continued) and S1A

## Slide 9
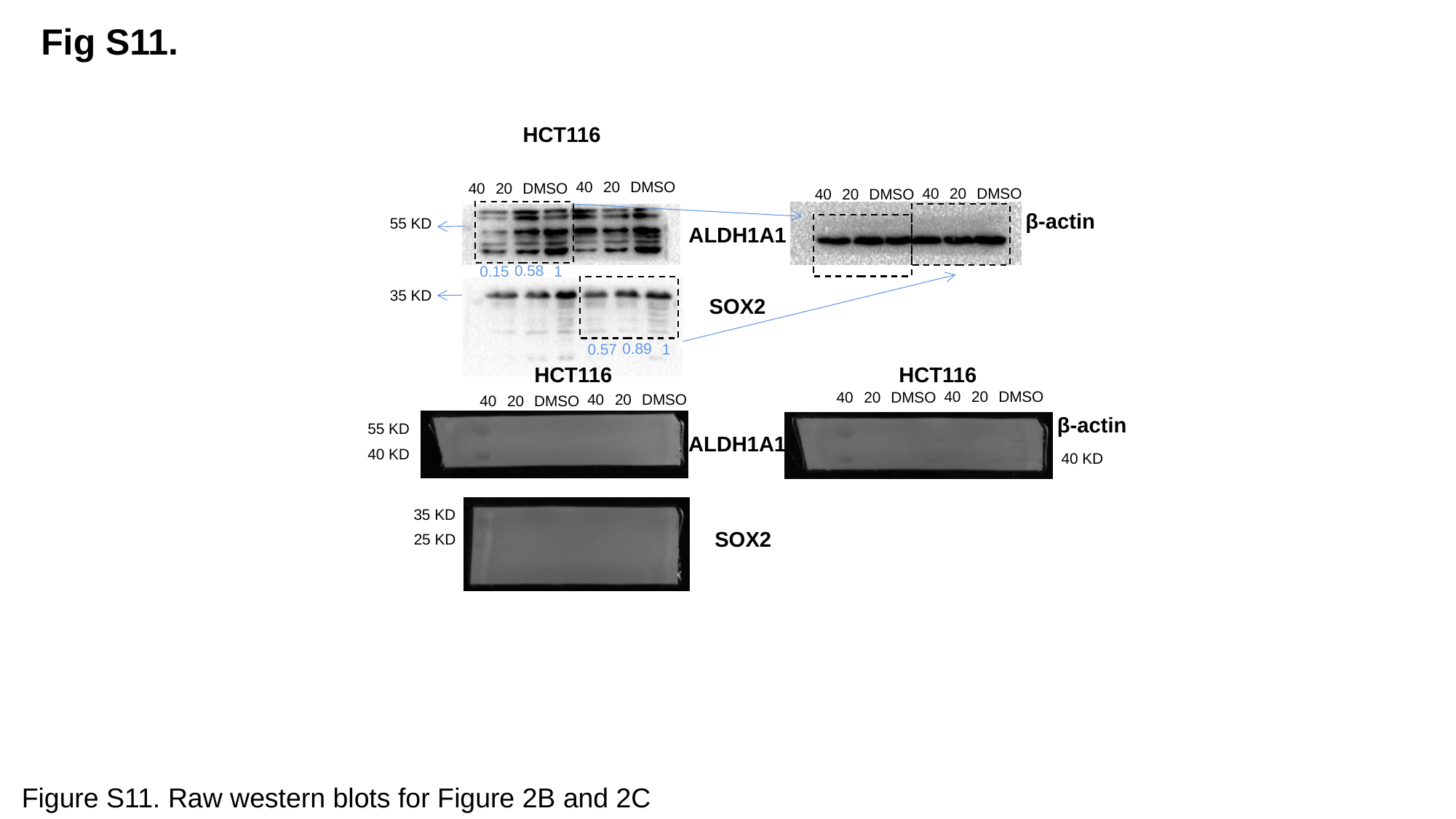

Fig S11.
HCT116
40
20
DMSO
40
20
DMSO
40
20
DMSO
40
20
DMSO
β-actin
55 KD
ALDH1A1
0.58
0.15
1
35 KD
SOX2
0.89
0.57
1
HCT116
HCT116
40
20
DMSO
40
20
DMSO
40
20
DMSO
40
20
DMSO
β-actin
55 KD
ALDH1A1
40 KD
40 KD
35 KD
SOX2
25 KD
Figure S11. Raw western blots for Figure 2B and 2C

## Slide 10
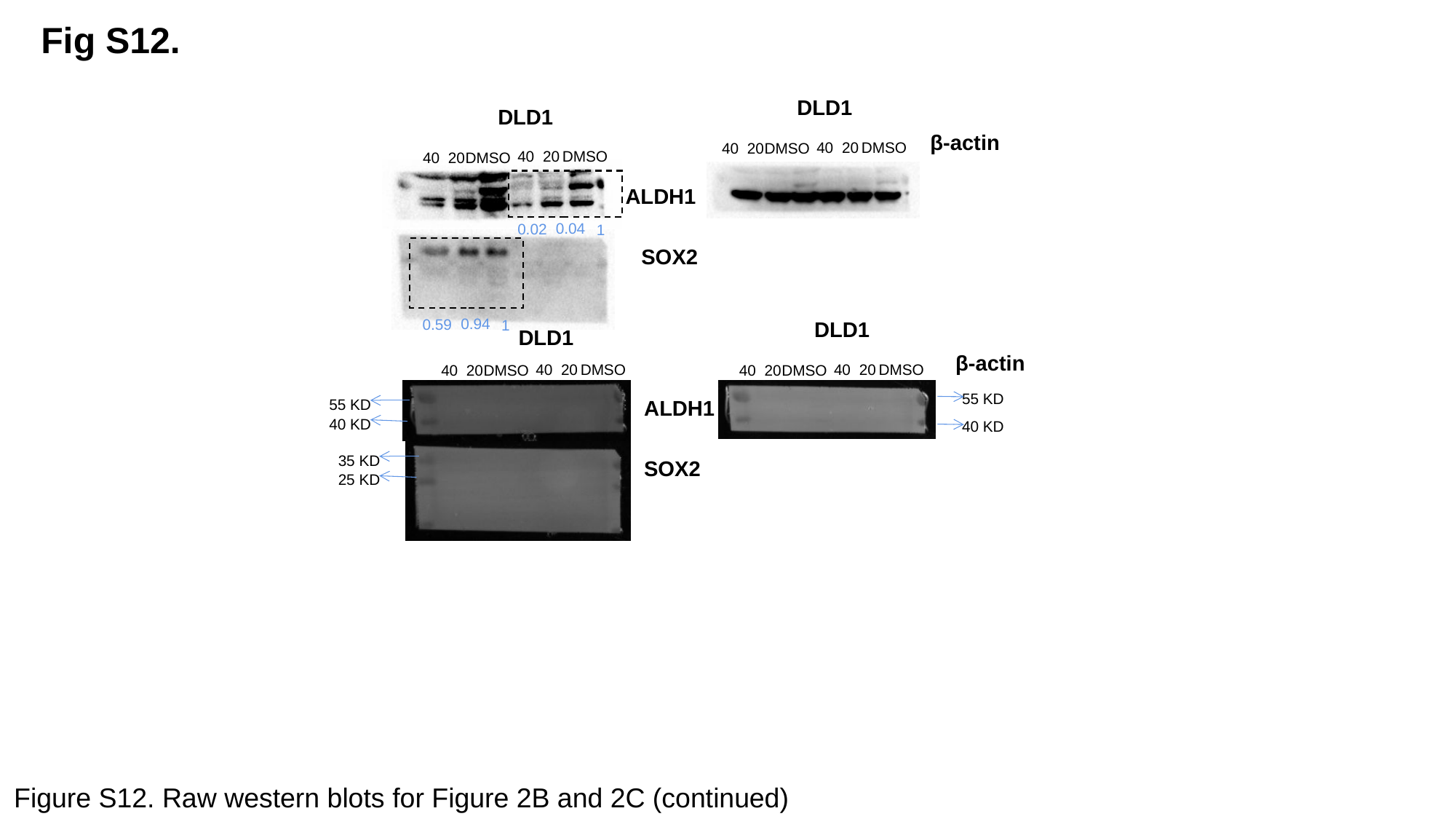

Fig S12.
DLD1
DLD1
β-actin
40
20
DMSO
40
20
DMSO
40
20
DMSO
40
20
DMSO
ALDH1
0.04
0.02
1
SOX2
0.94
0.59
1
DLD1
DLD1
β-actin
40
20
DMSO
40
20
DMSO
40
20
DMSO
40
20
DMSO
55 KD
55 KD
ALDH1
40 KD
40 KD
35 KD
SOX2
25 KD
Figure S12. Raw western blots for Figure 2B and 2C (continued)

## Slide 11
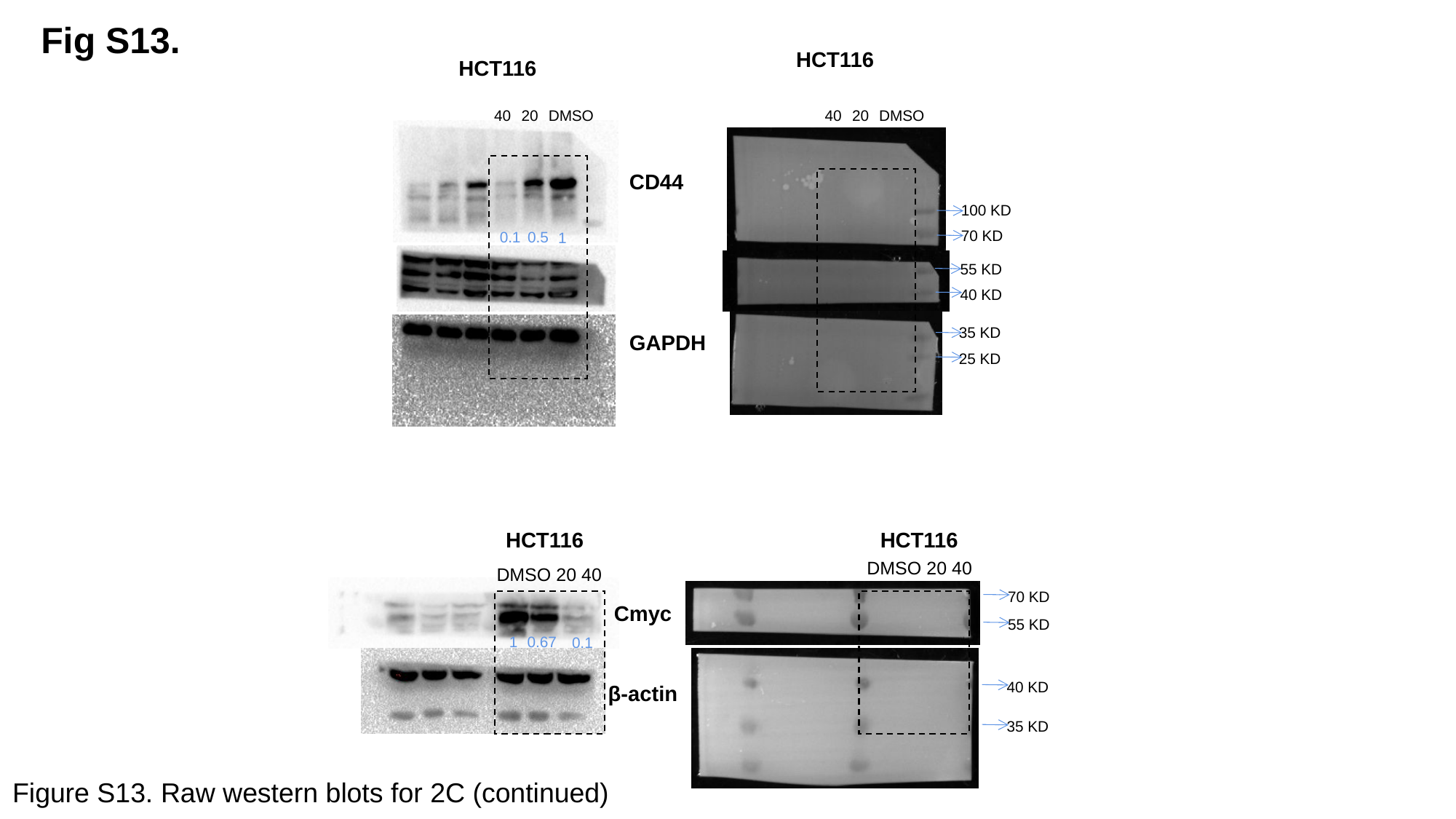

Fig S13.
HCT116
HCT116
40
20
DMSO
40
20
DMSO
CD44
100 KD
70 KD
0.1
0.5
1
55 KD
40 KD
35 KD
GAPDH
25 KD
HCT116
HCT116
DMSO 20 40
DMSO 20 40
70 KD
Cmyc
55 KD
1
0.67
0.1
40 KD
β-actin
35 KD
Figure S13. Raw western blots for 2C (continued)

## Slide 12
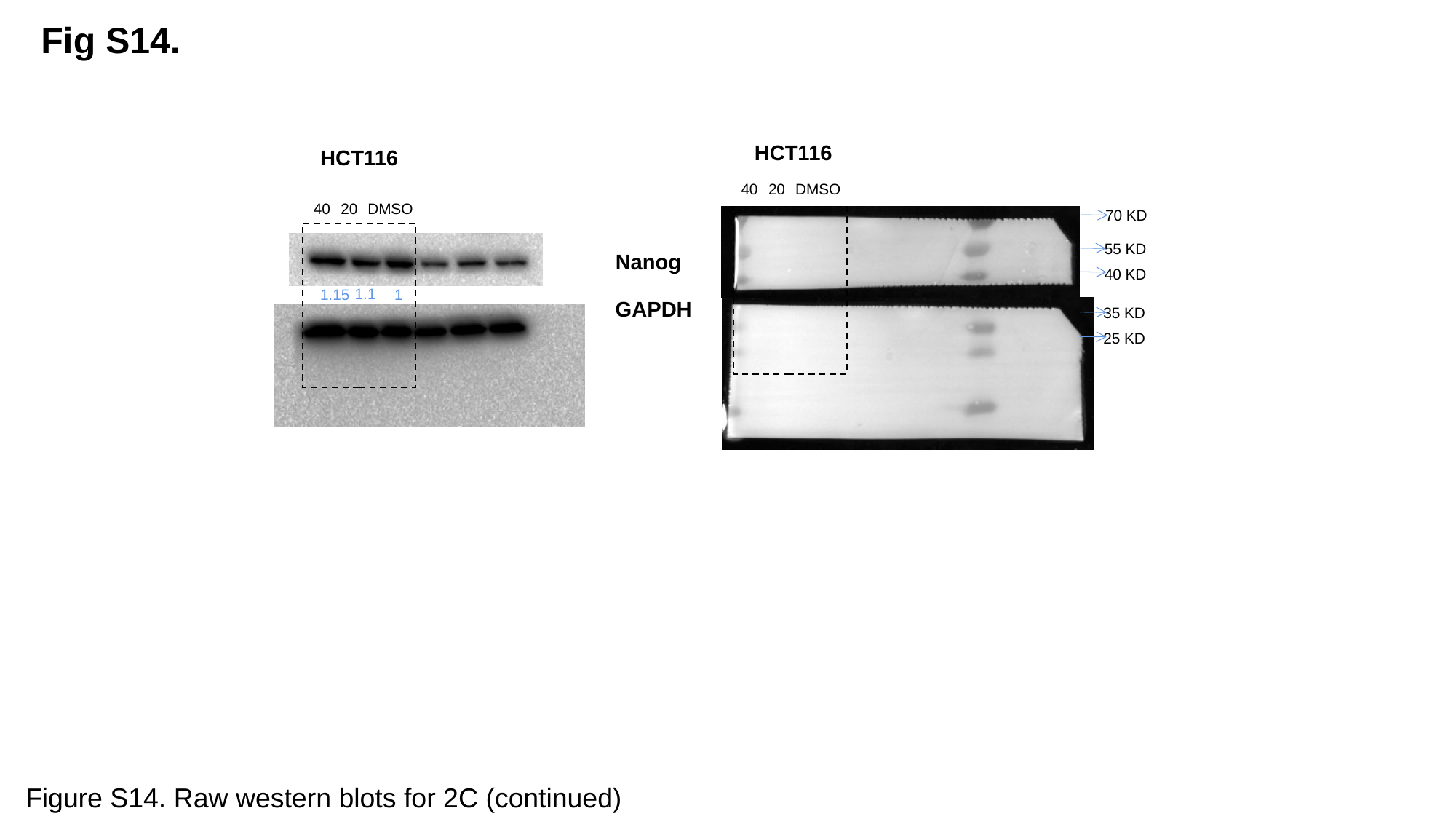

Fig S14.
HCT116
HCT116
40
20
DMSO
40
20
DMSO
70 KD
55 KD
Nanog
40 KD
1.1
1.15
1
GAPDH
35 KD
25 KD
Figure S14. Raw western blots for 2C (continued)

## Slide 13
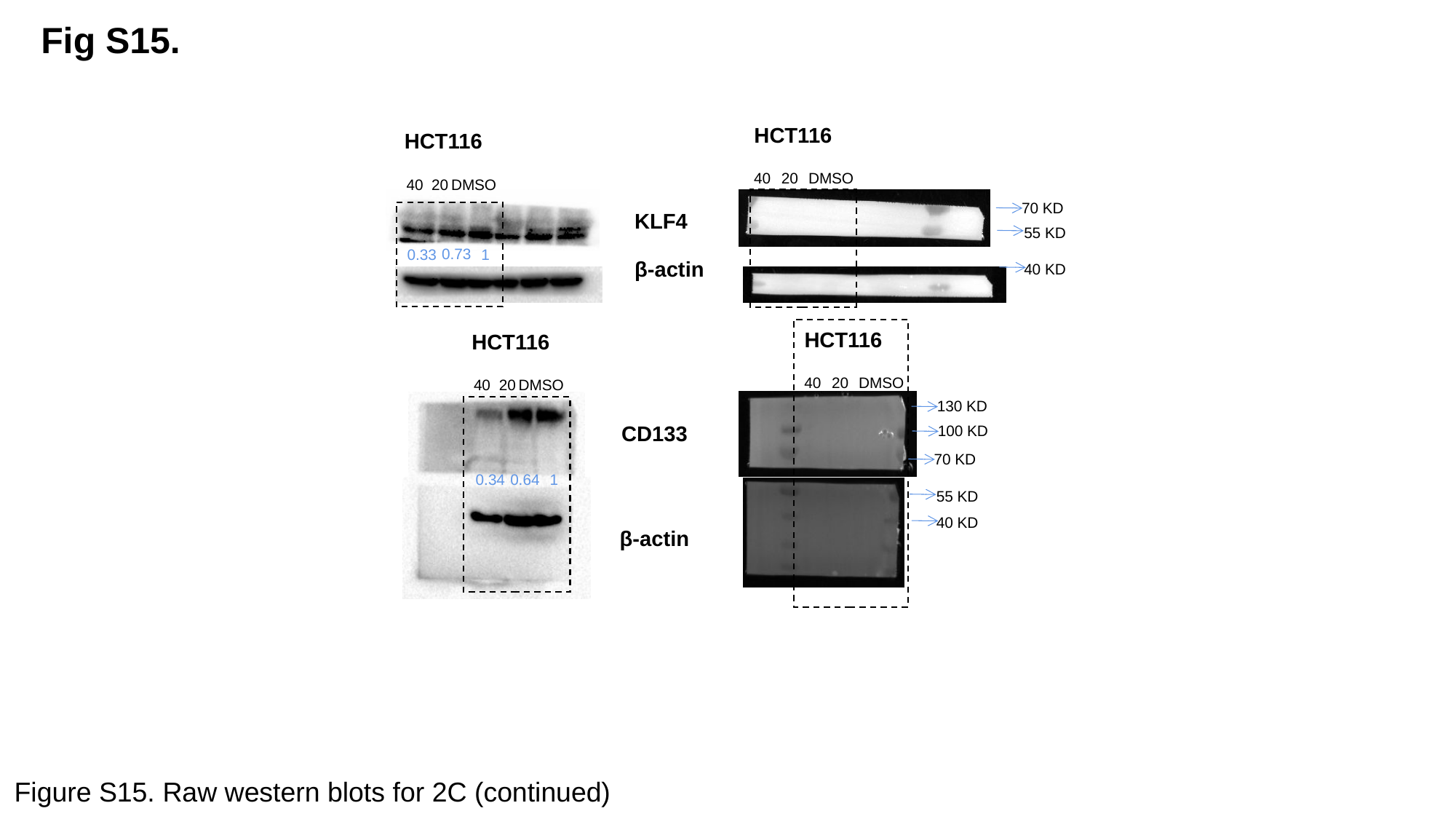

Fig S15.
HCT116
HCT116
40
20
DMSO
40
20
DMSO
70 KD
KLF4
55 KD
0.73
0.33
1
β-actin
40 KD
HCT116
HCT116
40
20
DMSO
40
20
DMSO
130 KD
CD133
100 KD
70 KD
0.64
0.34
1
55 KD
40 KD
β-actin
Figure S15. Raw western blots for 2C (continued)

## Slide 14
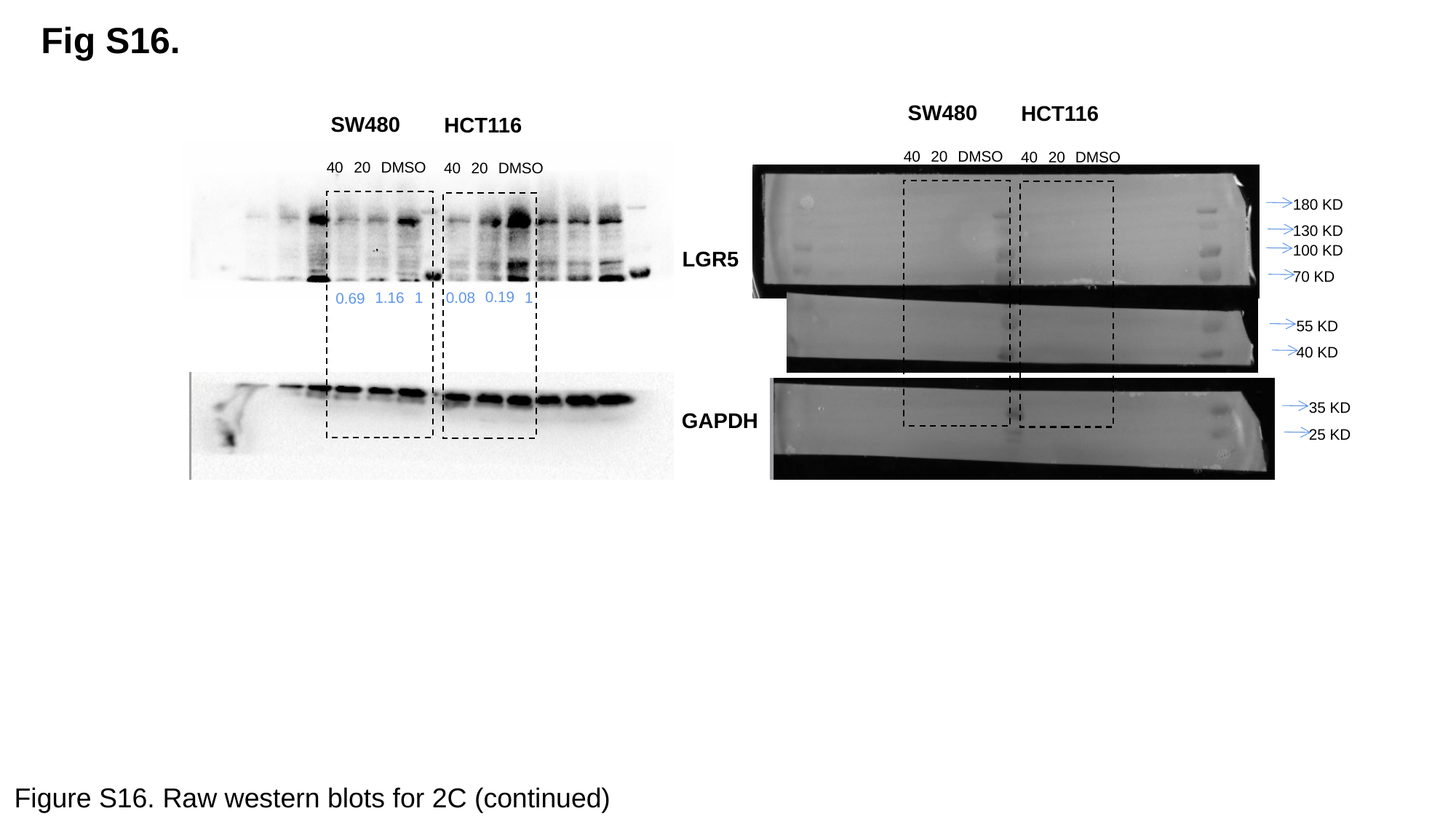

Fig S16.
SW480
HCT116
SW480
HCT116
40
20
DMSO
40
20
DMSO
40
20
DMSO
40
20
DMSO
180 KD
130 KD
100 KD
LGR5
70 KD
0.19
1.16
1
0.08
1
0.69
55 KD
40 KD
35 KD
GAPDH
25 KD
Figure S16. Raw western blots for 2C (continued)

## Slide 15
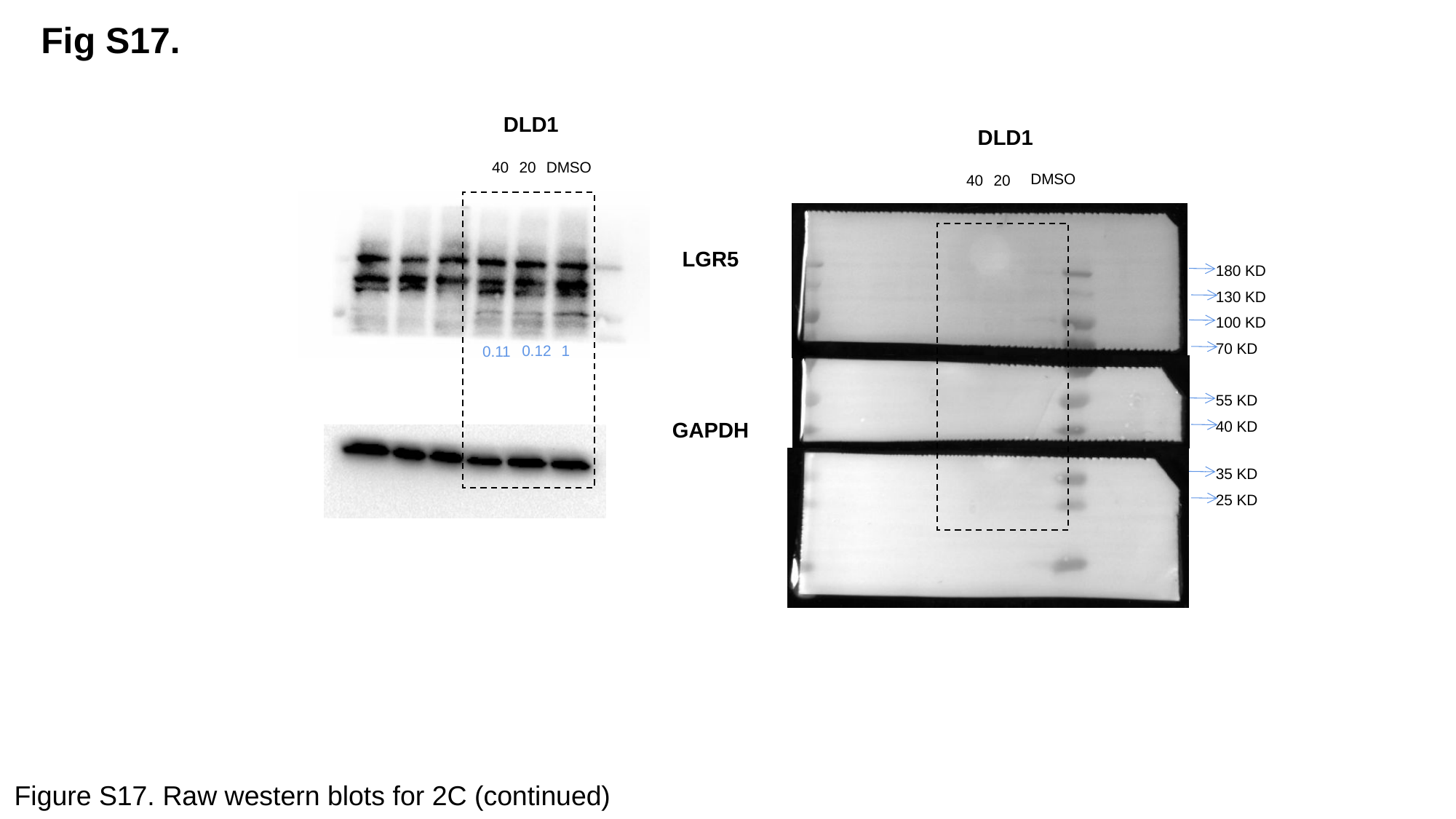

Fig S17.
DLD1
DLD1
40
20
DMSO
DMSO
40
20
LGR5
180 KD
130 KD
100 KD
70 KD
0.12
1
0.11
55 KD
GAPDH
40 KD
35 KD
25 KD
Figure S17. Raw western blots for 2C (continued)

## Slide 16
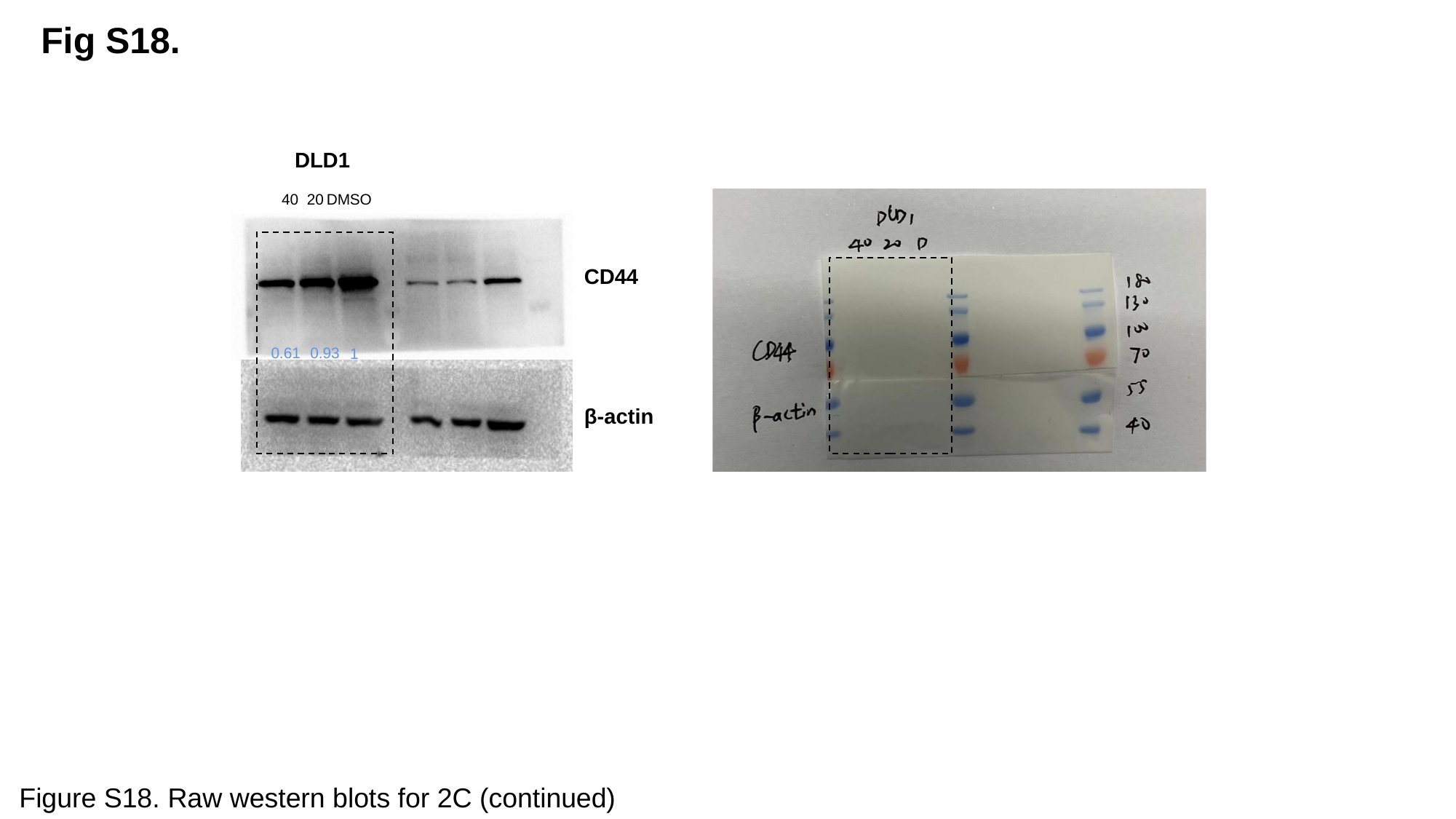

Fig S18.
DLD1
40
20
DMSO
CD44
0.61
0.93
1
β-actin
Figure S18. Raw western blots for 2C (continued)

## Slide 17
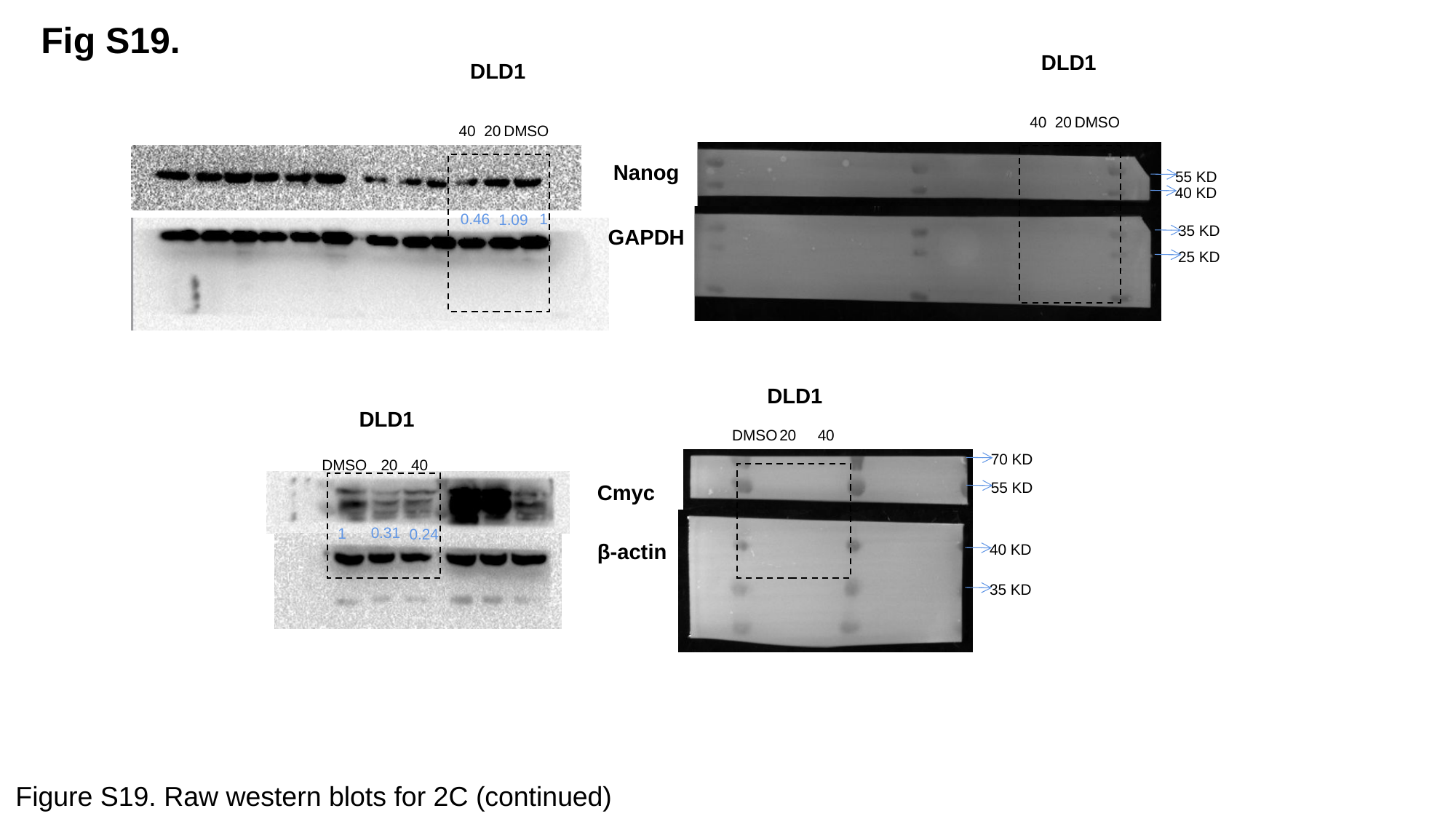

Fig S19.
DLD1
DLD1
40
20
DMSO
40
20
DMSO
Nanog
55 KD
40 KD
1
0.46
1.09
35 KD
GAPDH
25 KD
DLD1
DLD1
DMSO
20
40
70 KD
20
40
DMSO
55 KD
Cmyc
0.31
1
0.24
β-actin
40 KD
35 KD
Figure S19. Raw western blots for 2C (continued)

## Slide 18
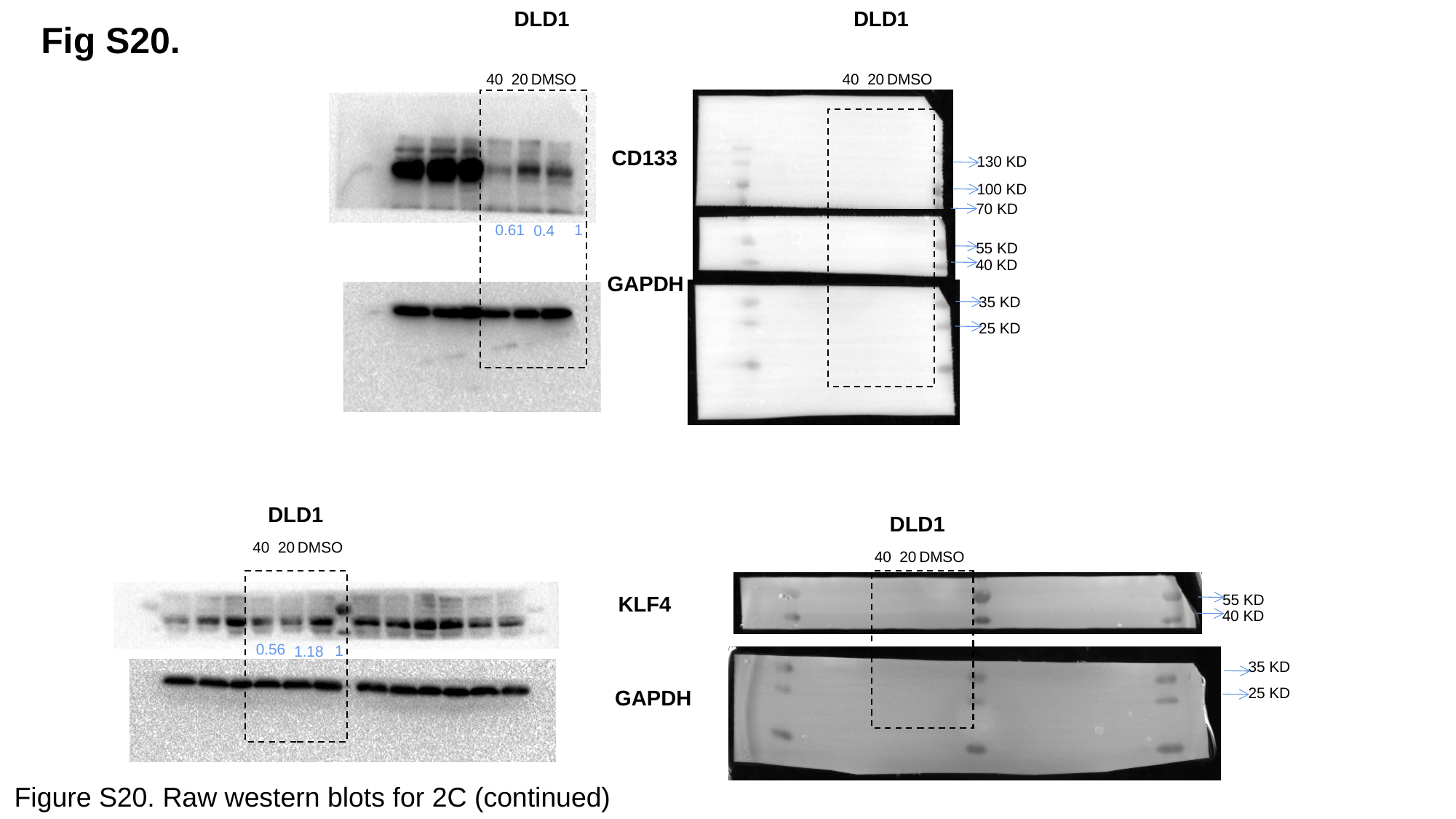

DLD1
DLD1
40
20
DMSO
40
20
DMSO
CD133
130 KD
100 KD
70 KD
1
0.61
0.4
55 KD
40 KD
GAPDH
35 KD
25 KD
Fig S20.
DLD1
DLD1
40
20
DMSO
40
20
DMSO
55 KD
KLF4
40 KD
0.56
1
1.18
35 KD
25 KD
GAPDH
Figure S20. Raw western blots for 2C (continued)

## Slide 19
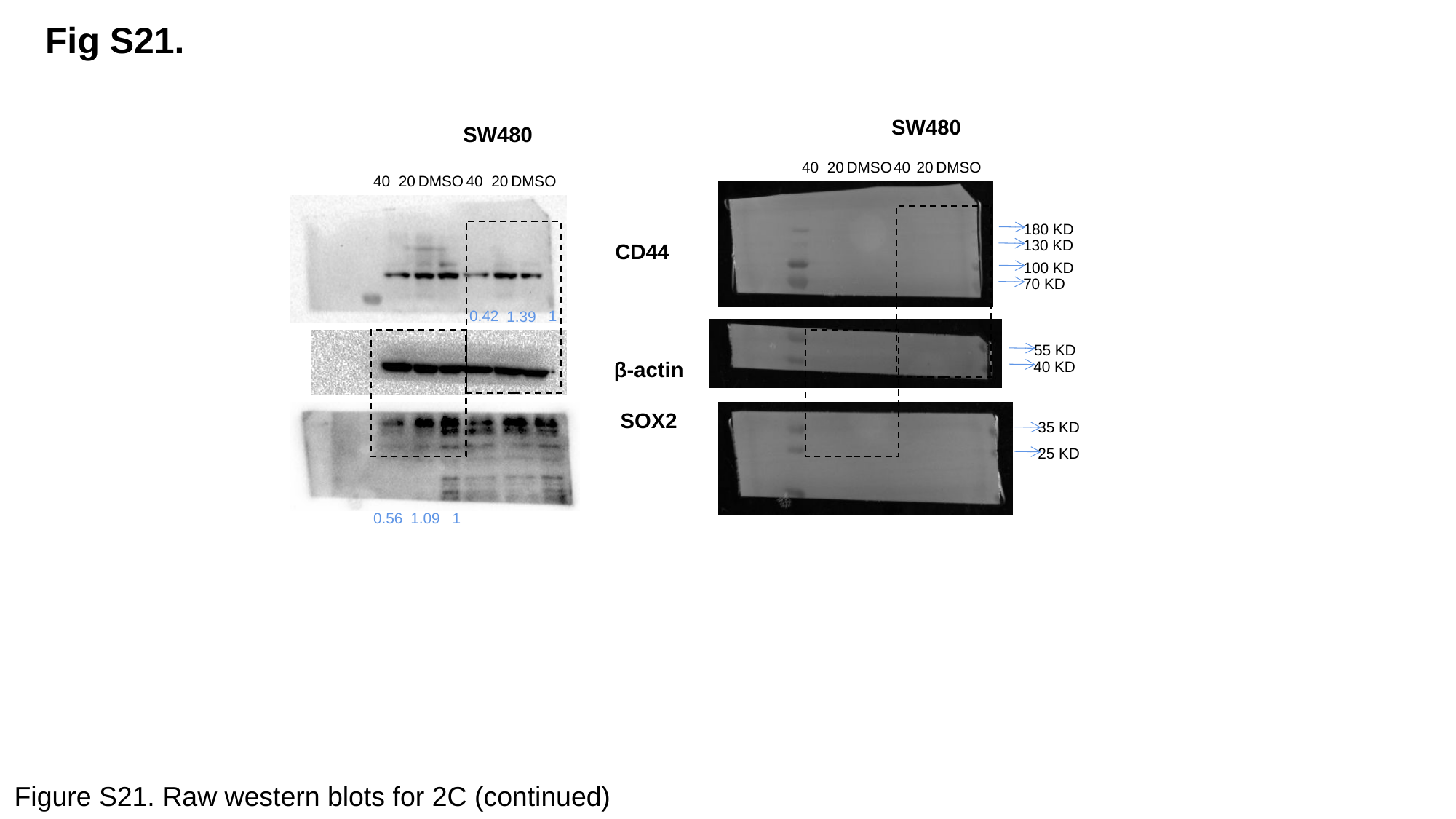

Fig S21.
SW480
SW480
40
20
DMSO
40
20
DMSO
40
20
DMSO
40
20
DMSO
180 KD
130 KD
CD44
100 KD
70 KD
1
0.42
1.39
55 KD
β-actin
40 KD
SOX2
35 KD
25 KD
1
0.56
1.09
Figure S21. Raw western blots for 2C (continued)

## Slide 20
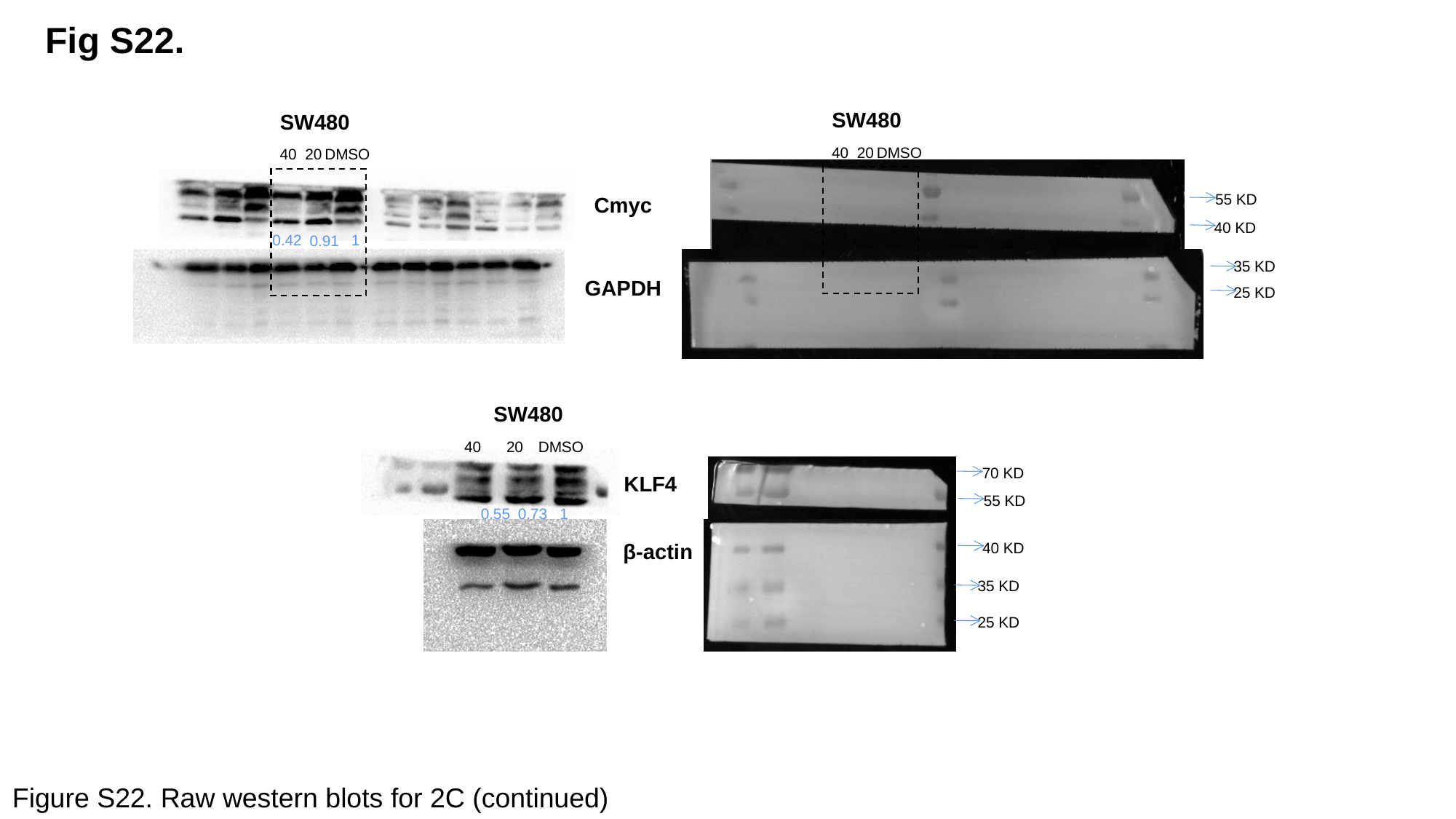

Fig S22.
SW480
SW480
40
20
DMSO
40
20
DMSO
55 KD
Cmyc
40 KD
1
0.42
0.91
35 KD
GAPDH
25 KD
SW480
40
20
DMSO
70 KD
KLF4
55 KD
1
0.55
0.73
β-actin
40 KD
35 KD
25 KD
Figure S22. Raw western blots for 2C (continued)

## Slide 21
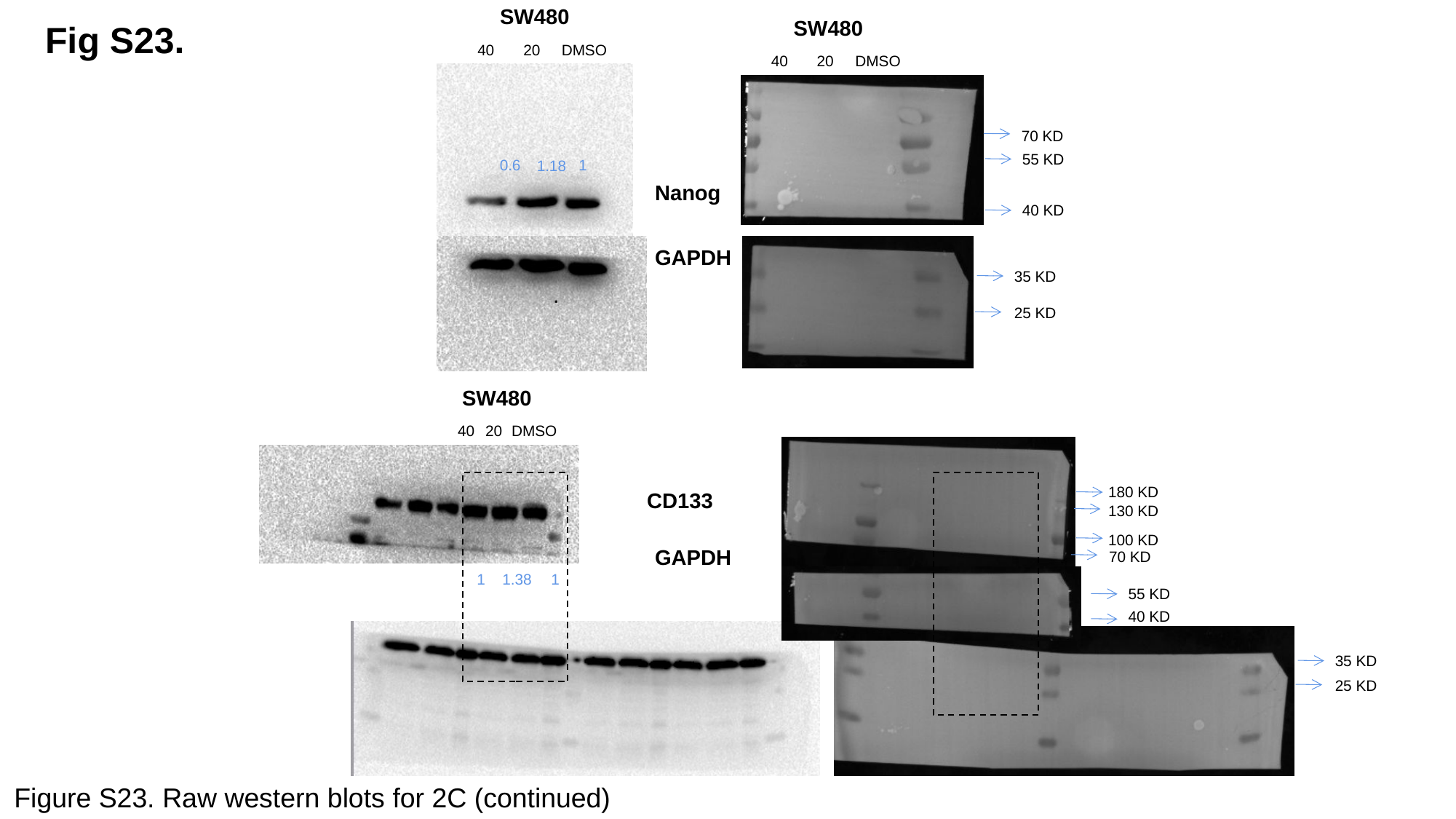

SW480
SW480
40
20
DMSO
40
20
DMSO
70 KD
55 KD
1
0.6
1.18
Nanog
40 KD
GAPDH
35 KD
25 KD
Fig S23.
SW480
40
20
DMSO
180 KD
CD133
130 KD
100 KD
GAPDH
70 KD
1
1
1.38
55 KD
40 KD
35 KD
25 KD
Figure S23. Raw western blots for 2C (continued)

## Slide 22
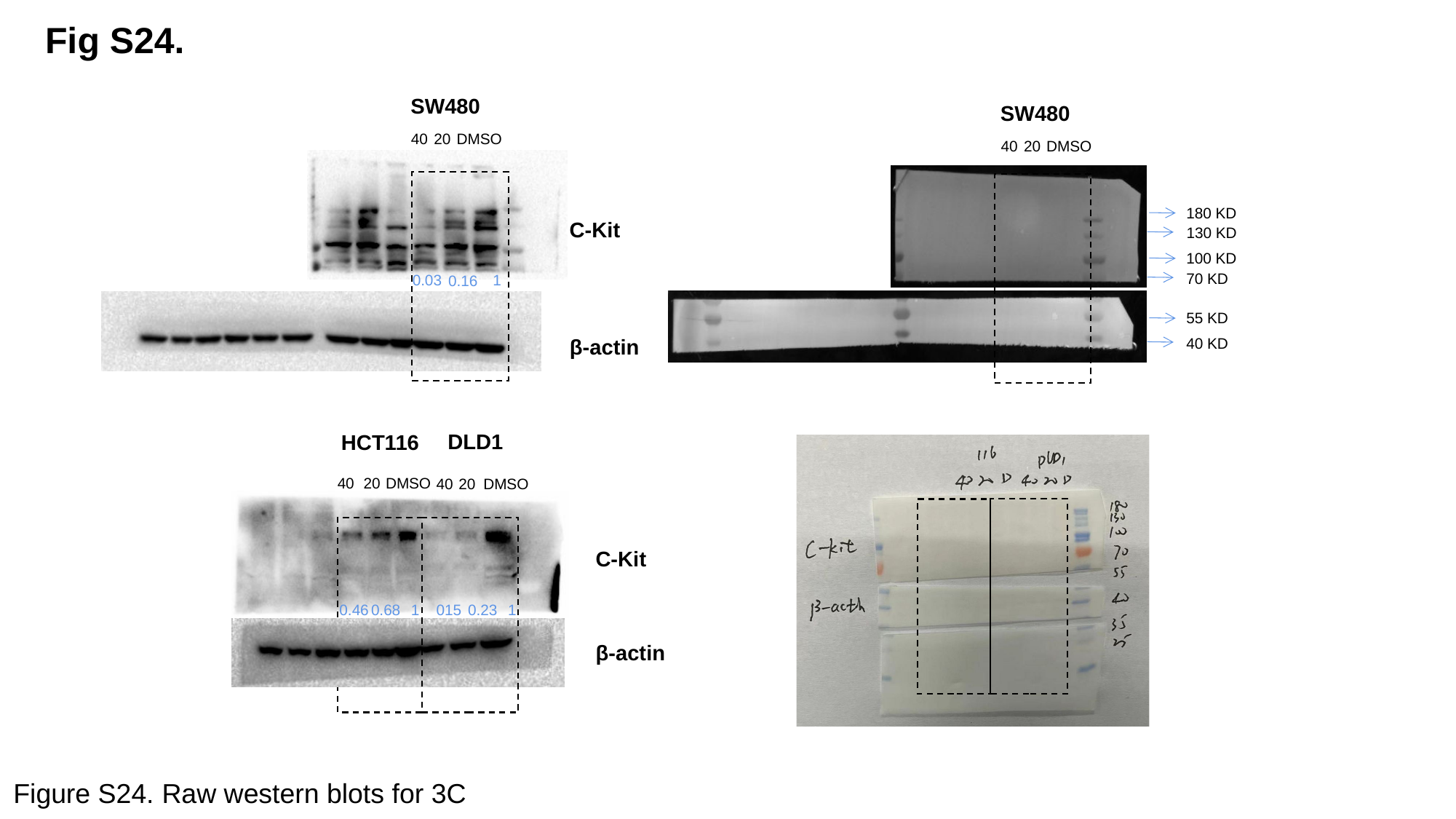

Fig S24.
SW480
SW480
40
20
DMSO
40
20
DMSO
180 KD
C-Kit
130 KD
100 KD
70 KD
0.03
1
0.16
55 KD
β-actin
40 KD
DLD1
HCT116
40
20
DMSO
40
20
DMSO
C-Kit
0.46
0.68
1
015
0.23
1
β-actin
Figure S24. Raw western blots for 3C

## Slide 23
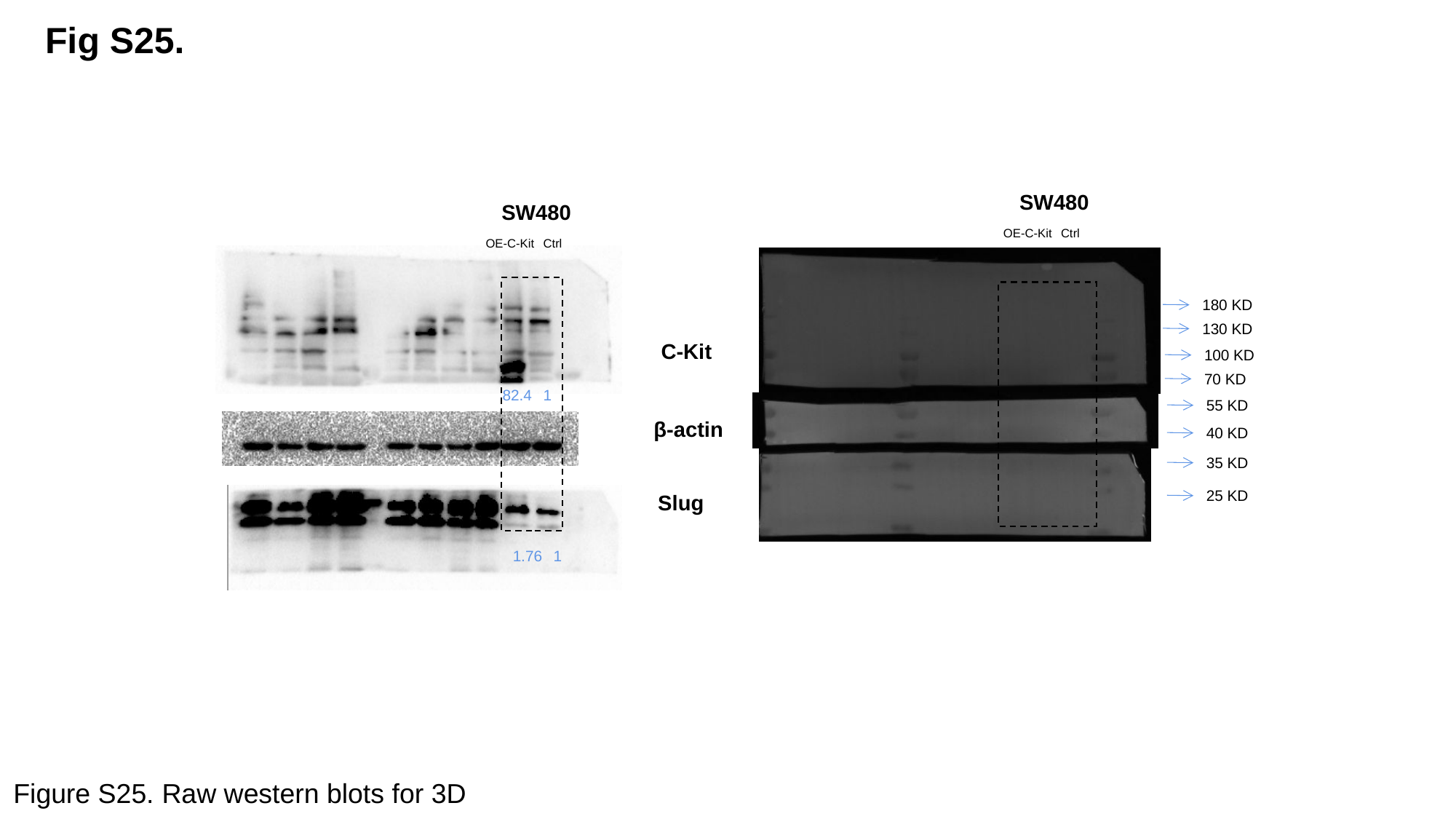

Fig S25.
SW480
SW480
OE-C-Kit
Ctrl
OE-C-Kit
Ctrl
180 KD
130 KD
C-Kit
100 KD
70 KD
82.4
1
55 KD
β-actin
40 KD
35 KD
25 KD
Slug
1.76
1
Figure S25. Raw western blots for 3D

## Slide 24
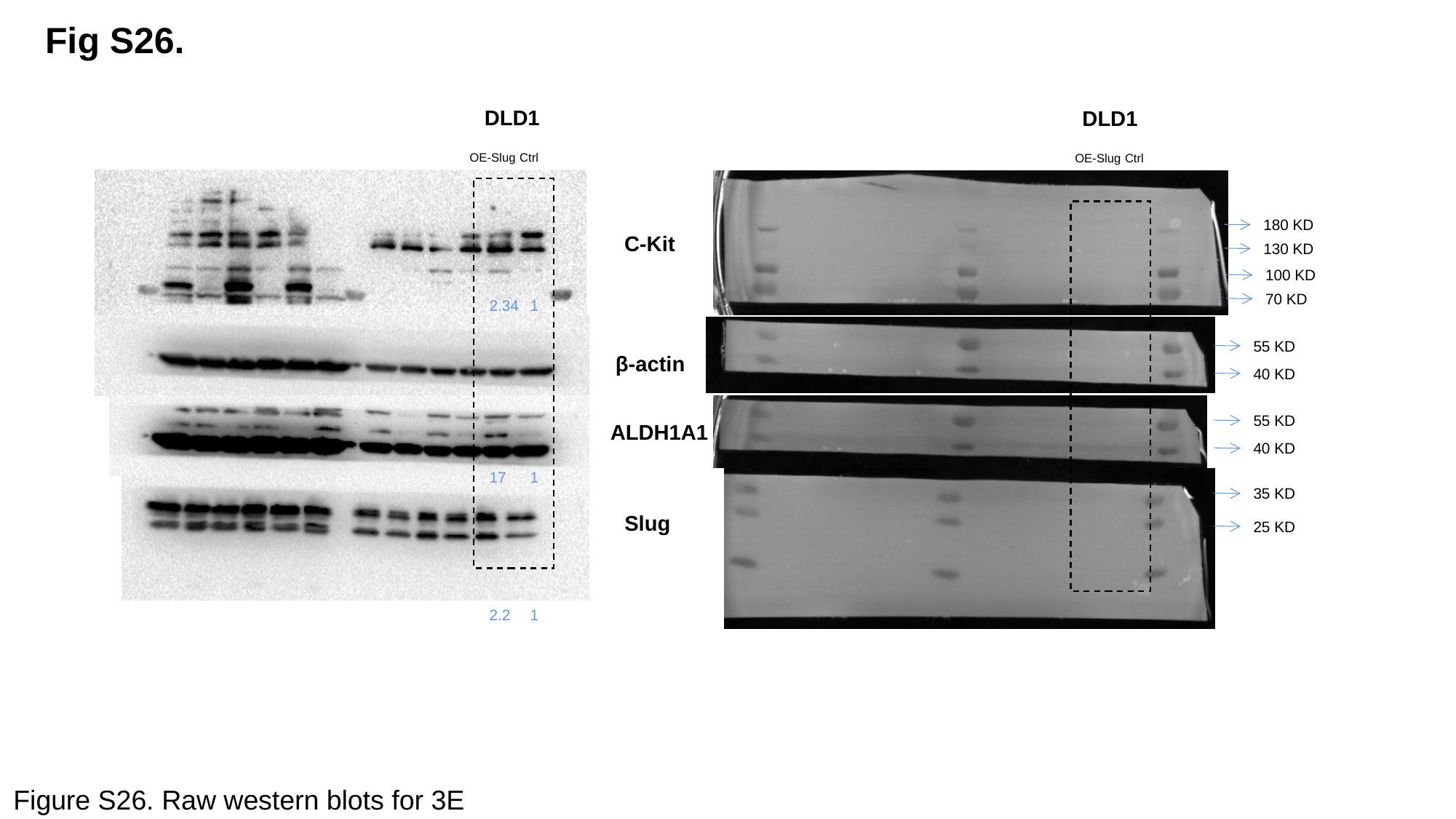

Fig S26.
DLD1
DLD1
OE-Slug
Ctrl
OE-Slug
Ctrl
180 KD
C-Kit
130 KD
100 KD
70 KD
2.34
1
55 KD
β-actin
40 KD
55 KD
ALDH1A1
40 KD
17
1
35 KD
Slug
25 KD
2.2
1
Figure S26. Raw western blots for 3E

## Slide 25
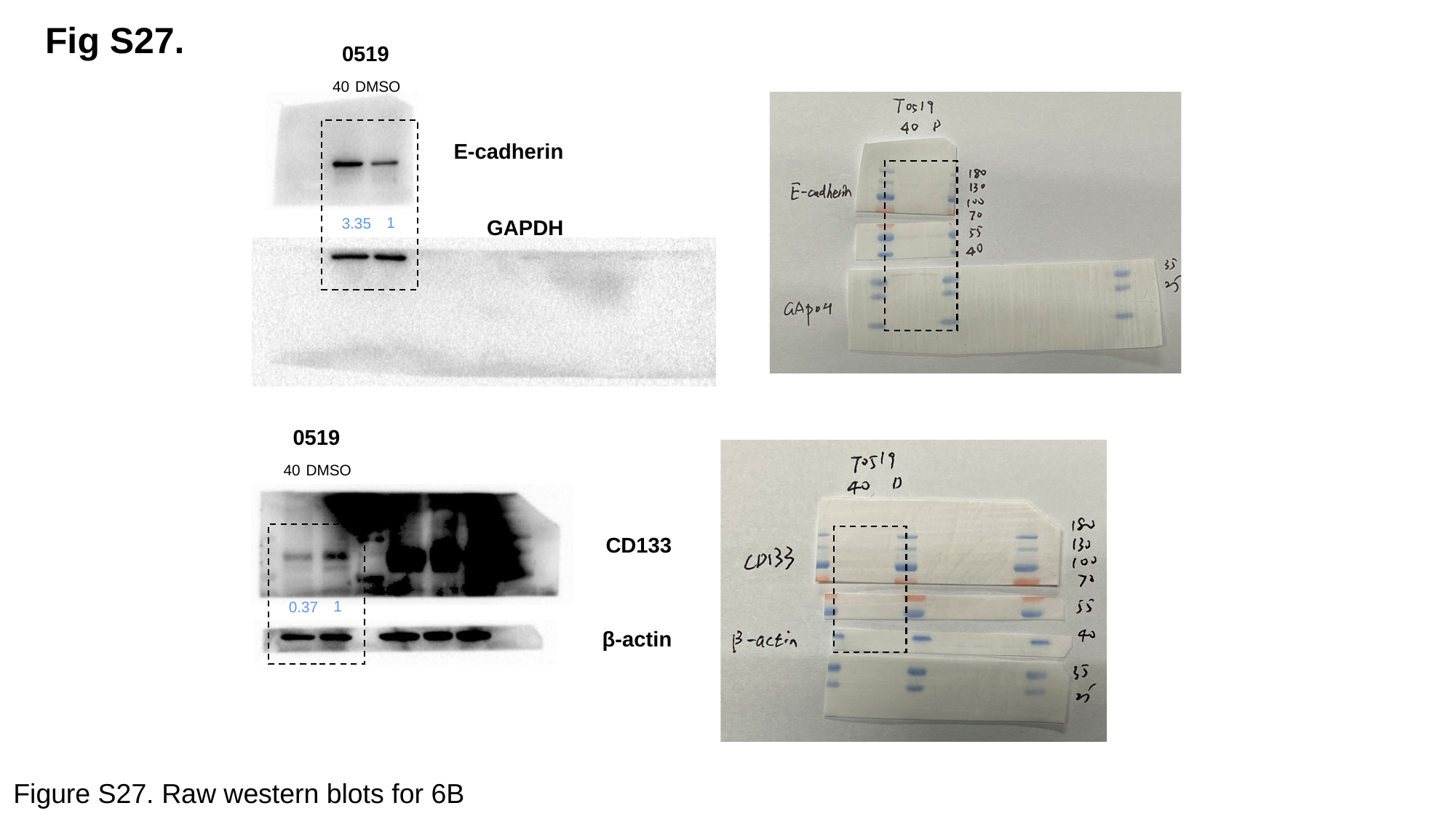

Fig S27.
0519
40
DMSO
E-cadherin
1
3.35
GAPDH
0519
40
DMSO
CD133
1
0.37
β-actin
Figure S27. Raw western blots for 6B

## Slide 26
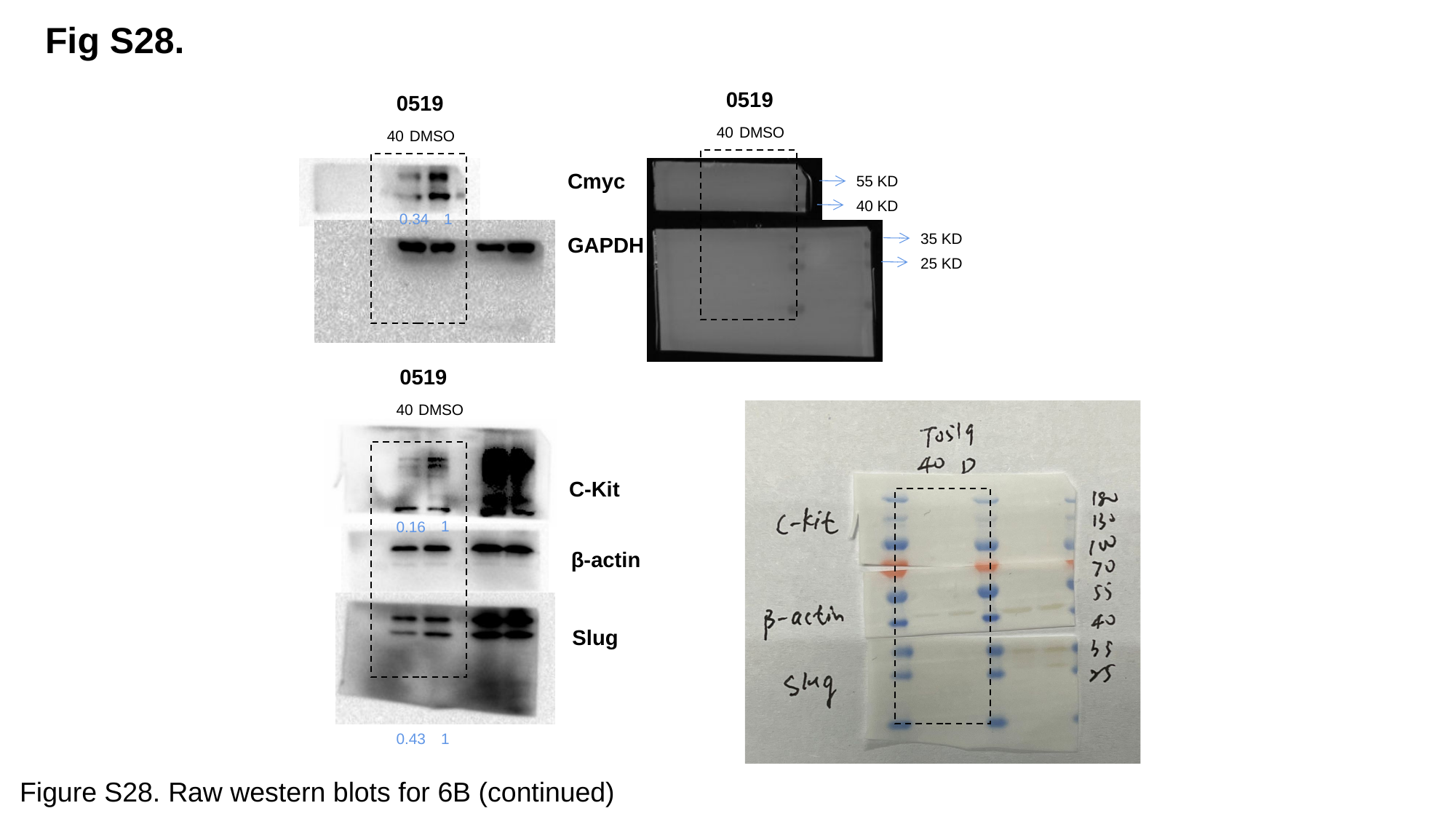

Fig S28.
0519
0519
40
DMSO
40
DMSO
Cmyc
55 KD
40 KD
1
0.34
35 KD
GAPDH
25 KD
0519
40
DMSO
C-Kit
1
0.16
β-actin
Slug
1
0.43
Figure S28. Raw western blots for 6B (continued)

## Slide 27
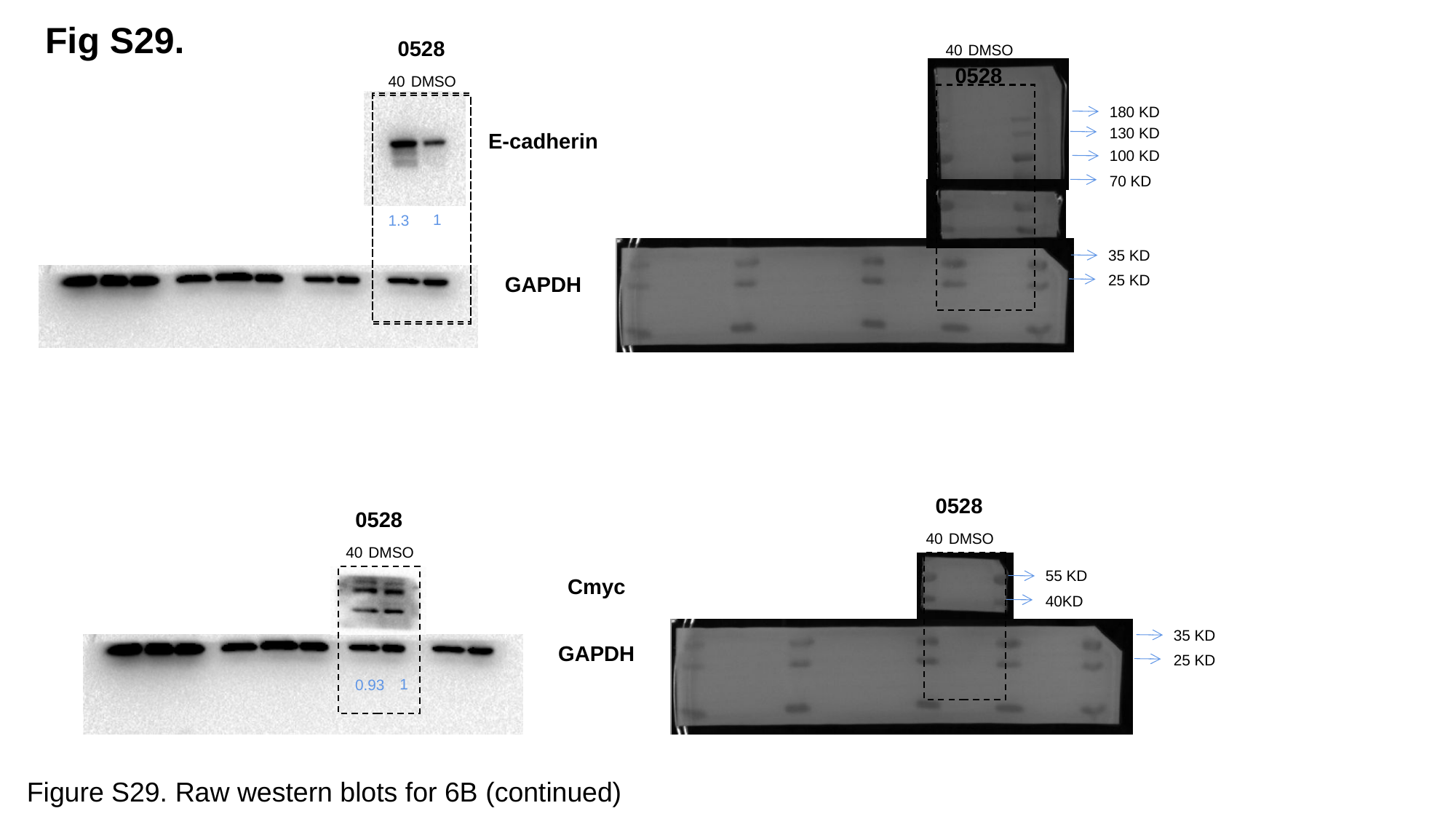

Fig S29.
0528
40
DMSO
0528
40
DMSO
180 KD
130 KD
E-cadherin
100 KD
70 KD
1
1.3
35 KD
GAPDH
25 KD
0528
0528
40
DMSO
40
DMSO
55 KD
Cmyc
40KD
35 KD
GAPDH
25 KD
1
0.93
Figure S29. Raw western blots for 6B (continued)

## Slide 28
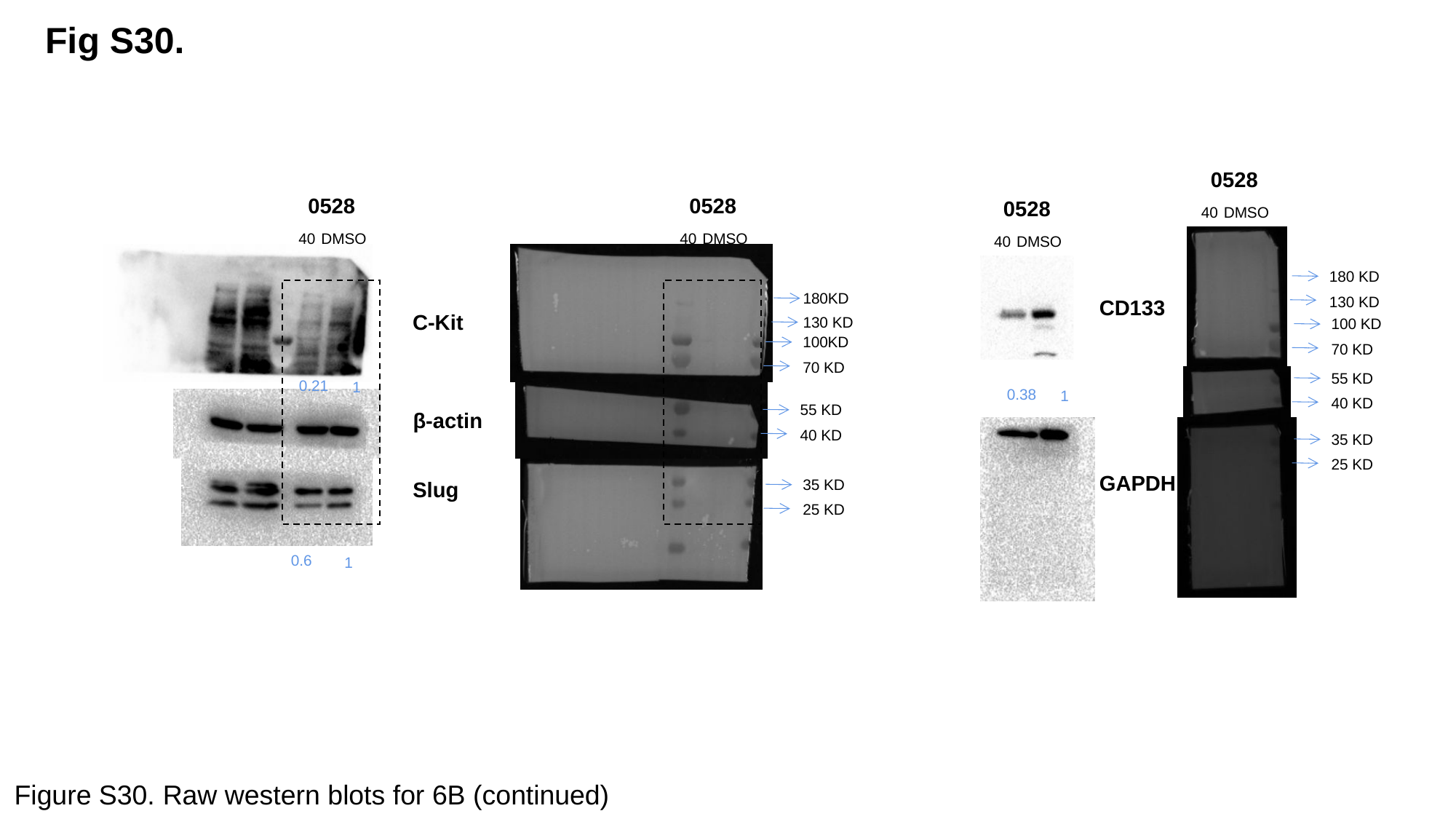

Fig S30.
0528
0528
40
DMSO
40
DMSO
180 KD
130 KD
CD133
100 KD
70 KD
55 KD
0.38
1
40 KD
35 KD
25 KD
GAPDH
0528
0528
40
DMSO
40
DMSO
180KD
C-Kit
130 KD
100KD
70 KD
0.21
1
55 KD
β-actin
40 KD
35 KD
Slug
25 KD
0.6
1
Figure S30. Raw western blots for 6B (continued)

## Slide 29
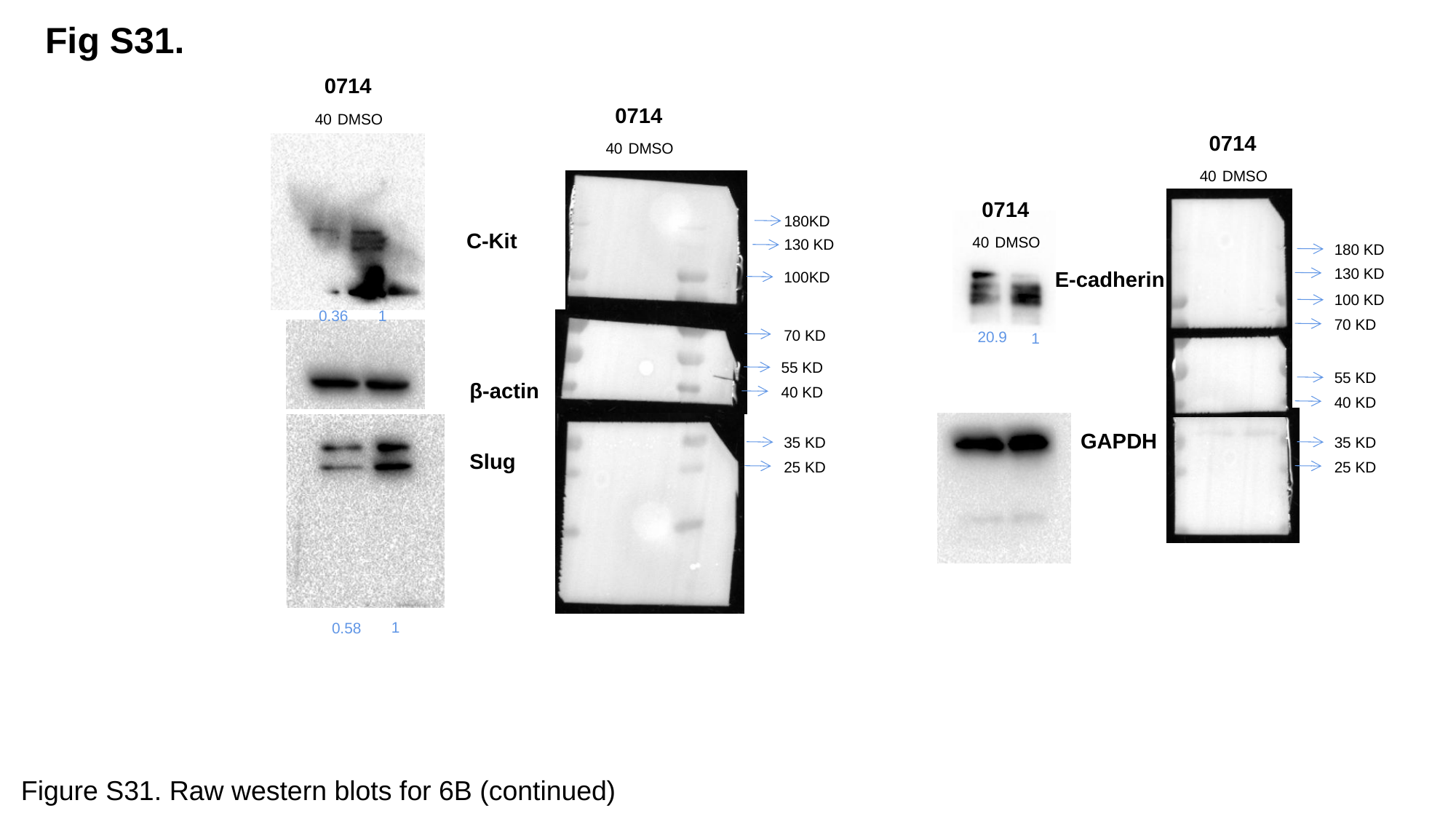

Fig S31.
0714
0714
40
DMSO
0714
40
DMSO
0714
40
DMSO
E-cadherin
20.9
1
GAPDH
40
DMSO
180KD
C-Kit
130 KD
180 KD
130 KD
100KD
100 KD
1
0.36
70 KD
70 KD
55 KD
55 KD
β-actin
40 KD
40 KD
35 KD
35 KD
Slug
25 KD
25 KD
1
0.58
Figure S31. Raw western blots for 6B (continued)

## Slide 30
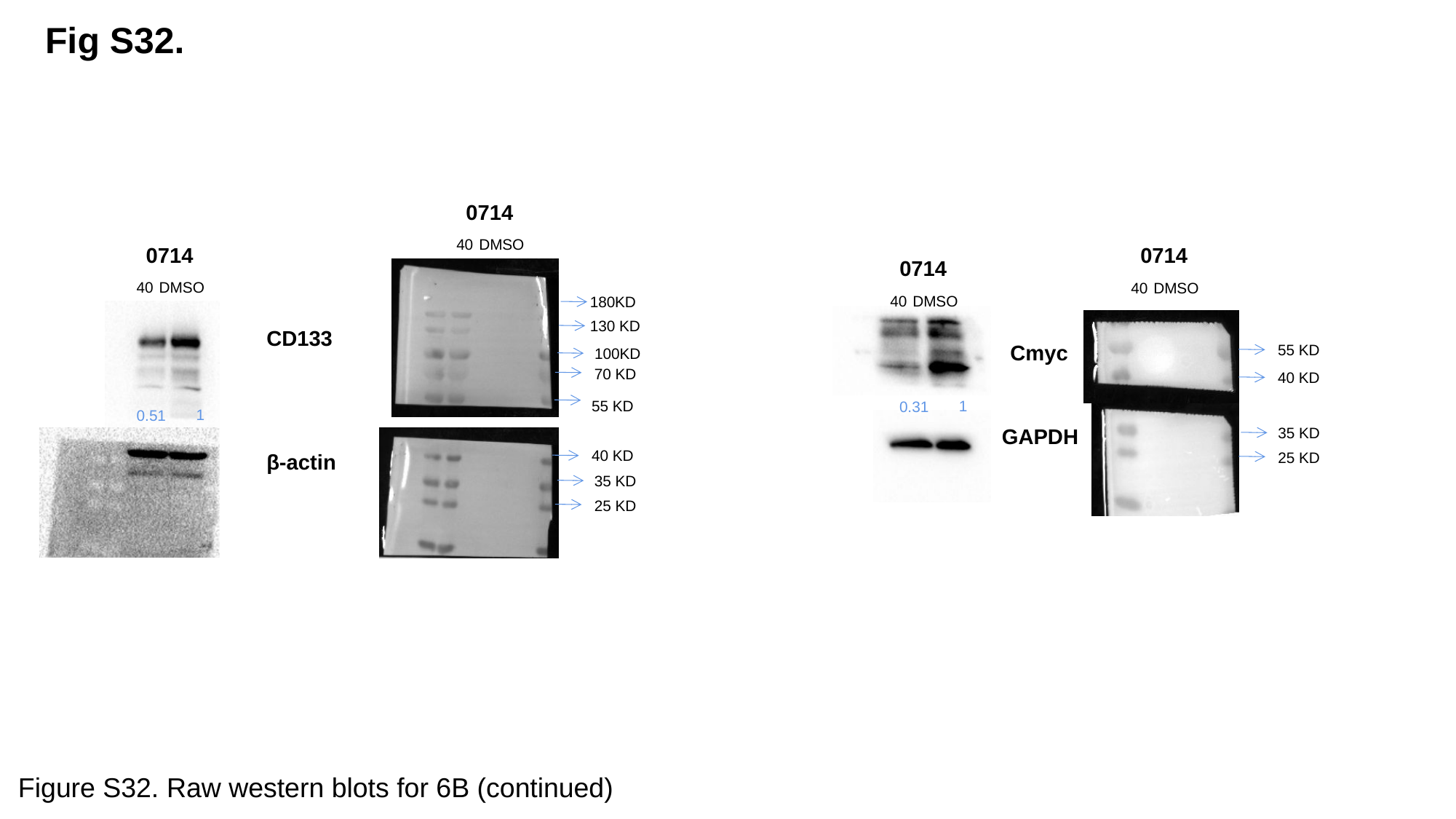

Fig S32.
0714
40
DMSO
0714
40
DMSO
180KD
130 KD
CD133
100KD
70 KD
55 KD
1
0.51
40 KD
β-actin
35 KD
25 KD
0714
0714
40
DMSO
40
DMSO
Cmyc
55 KD
40 KD
1
0.31
35 KD
GAPDH
25 KD
Figure S32. Raw western blots for 6B (continued)

## Slide 31
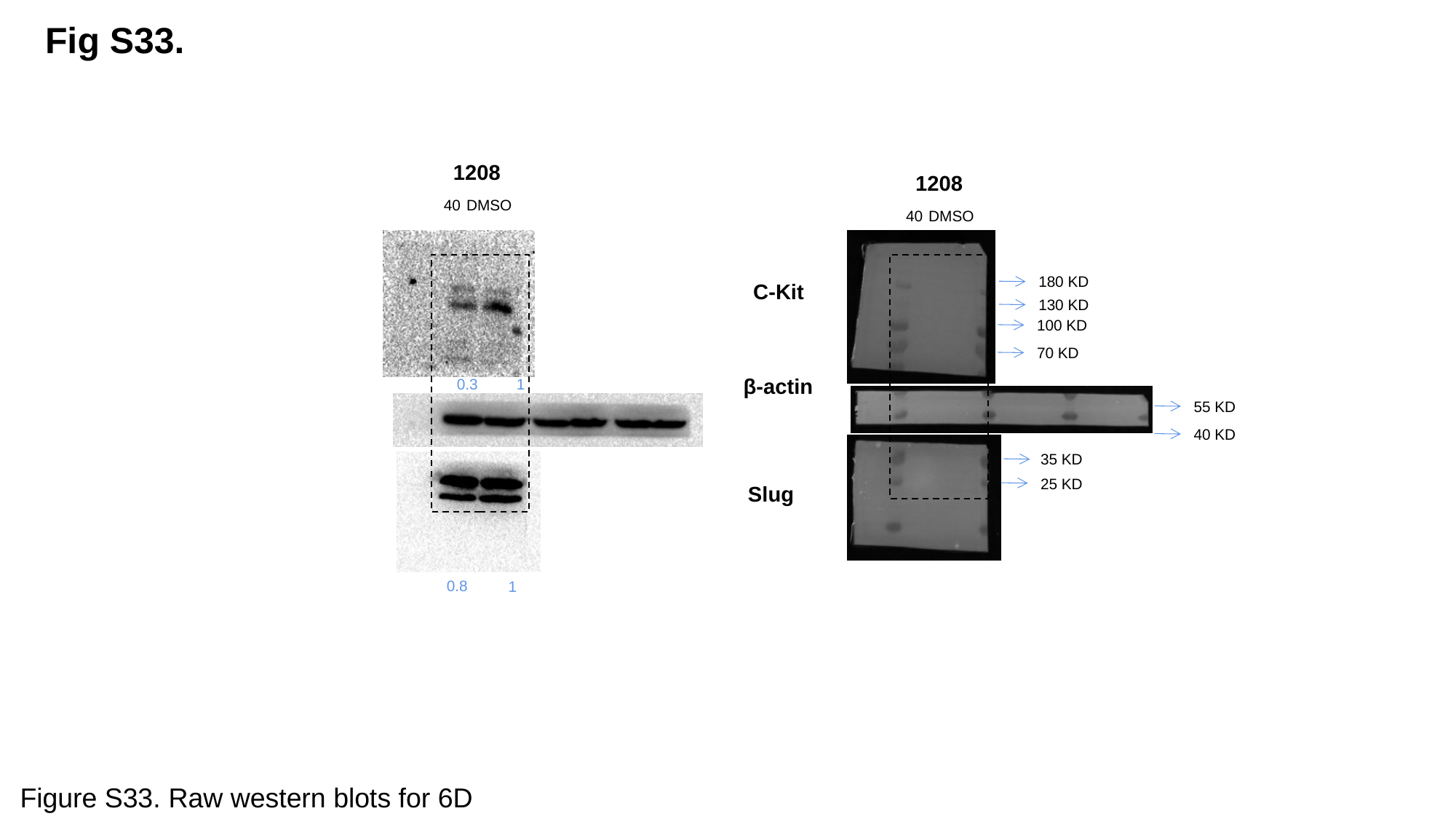

Fig S33.
1208
1208
40
DMSO
40
DMSO
180 KD
C-Kit
130 KD
100 KD
70 KD
β-actin
1
0.3
55 KD
40 KD
35 KD
25 KD
Slug
0.8
1
Figure S33. Raw western blots for 6D

## Slide 32
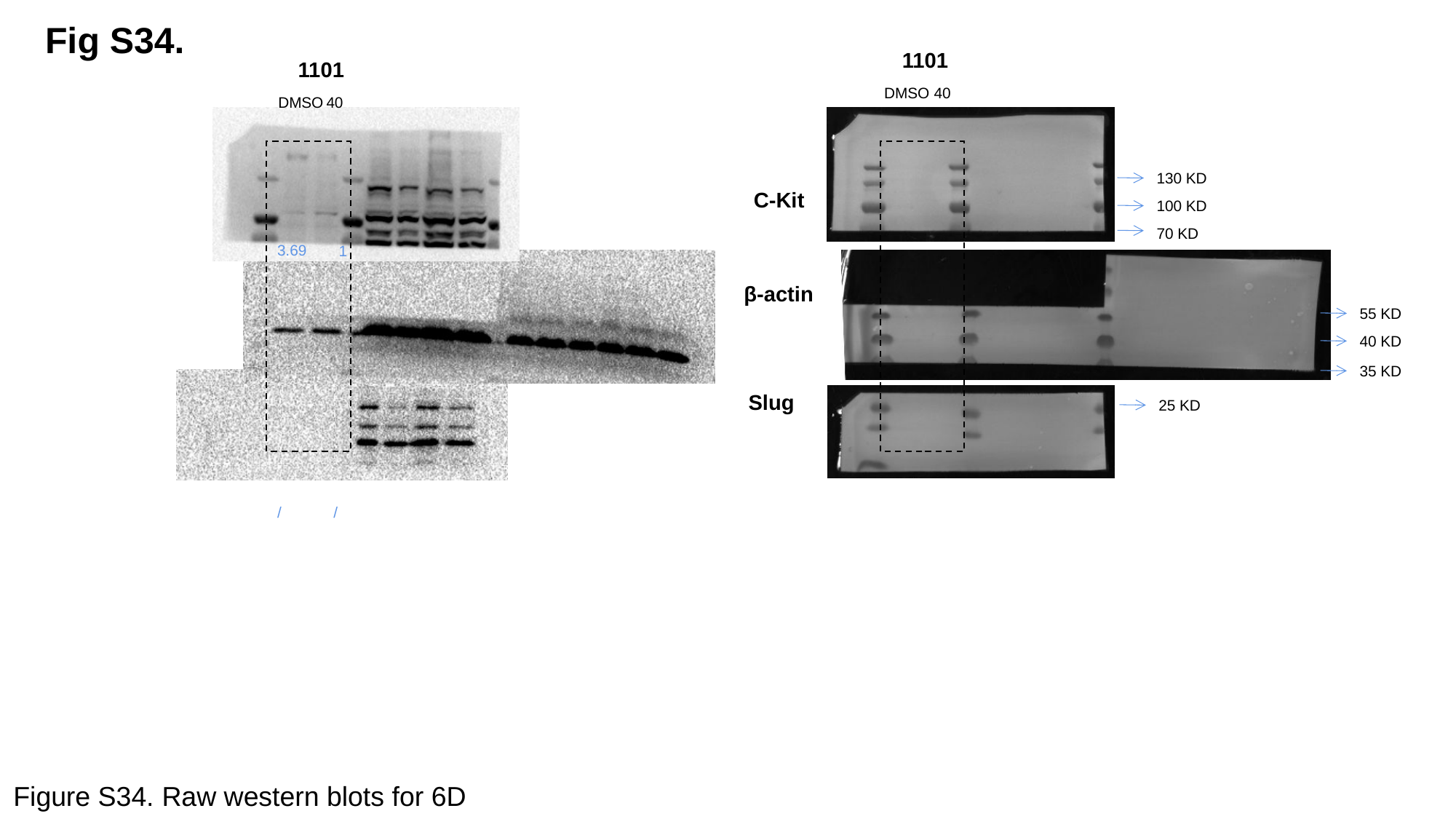

Fig S34.
1101
1101
DMSO
40
DMSO
40
130 KD
C-Kit
100 KD
70 KD
3.69
1
β-actin
55 KD
40 KD
35 KD
Slug
25 KD
/
/
Figure S34. Raw western blots for 6D

## Slide 33
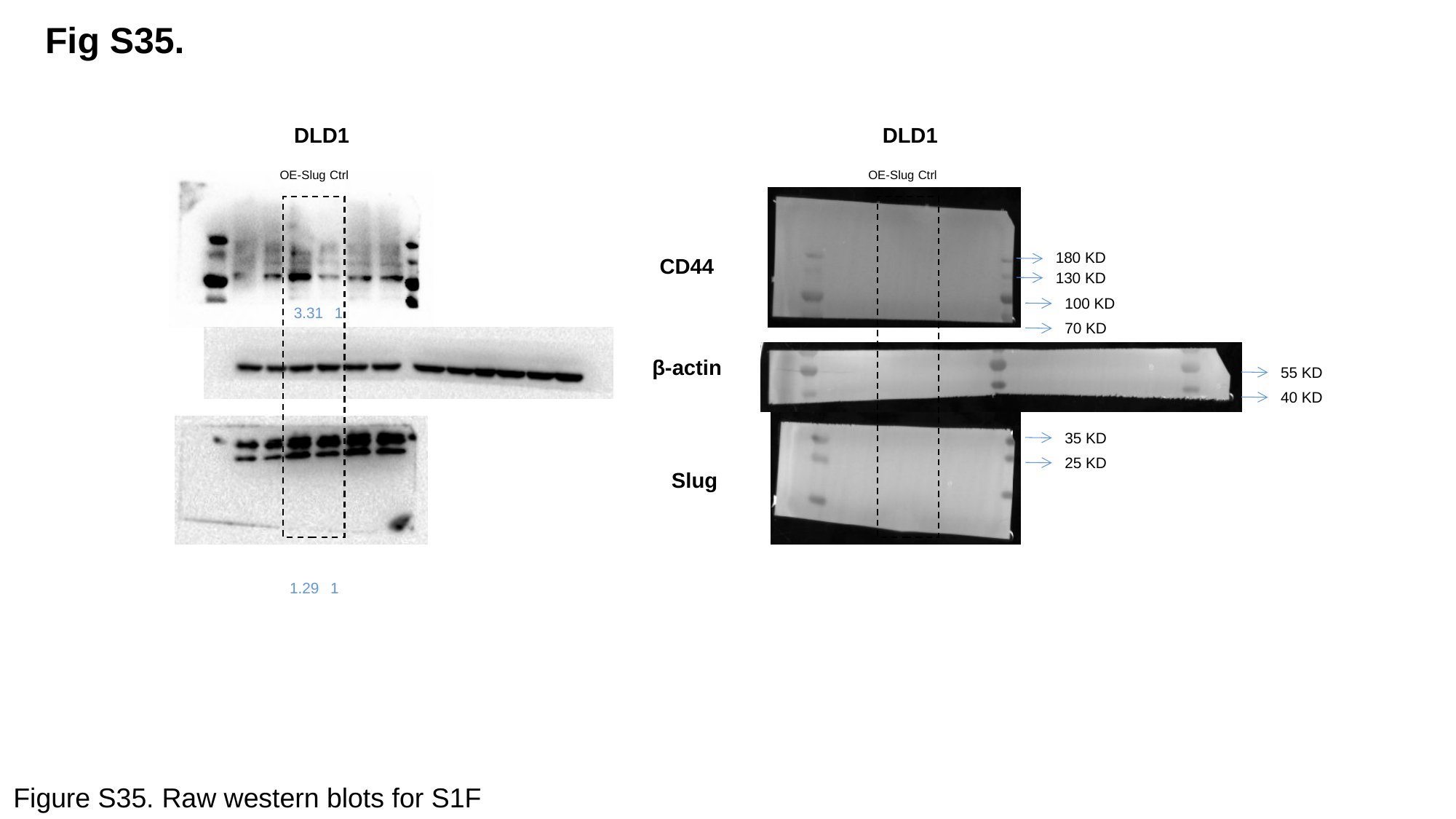

Fig S35.
DLD1
DLD1
OE-Slug
Ctrl
OE-Slug
Ctrl
180 KD
CD44
130 KD
100 KD
3.31
1
70 KD
β-actin
55 KD
40 KD
35 KD
25 KD
Slug
1.29
1
Figure S35. Raw western blots for S1F
